# The Antimicrobial and Antibiofilm In Vitro Activity of Liquid and Vapour Phases of Selected Essential Oils against *Staphylococcus aureus*

**DOI:** 10.3390/pathogens10091207

**Published:** 2021-09-17

**Authors:** Malwina Brożyna, Justyna Paleczny, Weronika Kozłowska, Grzegorz Chodaczek, Ruth Dudek-Wicher, Anna Felińczak, Joanna Gołębiewska, Agata Górniak, Adam Junka

**Affiliations:** 1 Department of Pharmaceutical Microbiology and Parasitology, Wroclaw Medical University, 50-556 Wroclaw, Poland; paleczny.justyna@gmail.com (J.P.); r.dudek.wicher@gmail.com (R.D.-W.); 2 Department of Pharmaceutical Biology, Wroclaw Medical University, 50-556 Wroclaw, Poland; weronika.kozlowska@umed.wroc.pl; 3 Bioimaging Laboratory, Łukasiewicz Research Network—PORT Polish Center for Technology Development, 54-066 Wroclaw, Poland; grzegorz.chodaczek@port.org.pl; 4 Department of Organisation and Management, Wroclaw Medical University, 51-618 Wroclaw, Poland; anna.felinczak@umed.wroc.pl; 5 Faculty of Medicine, Lazarski University, 02-662 Warsaw, Poland; joanna.golebiewska@lazarski.pl; 6 Laboratory of Elemental Analysis and Structural Research, Wroclaw Medical University, 50-556 Wroclaw, Poland; agata.gorniak@umed.wroc.pl

**Keywords:** *S. aureus*, biofilm, essential oils

## Abstract

The high resistance of staphylococcal biofilm against antibiotics and developing resistance against antiseptics induces a search for novel antimicrobial compounds. Due to acknowledged and/or alleged antimicrobial activity of EOs, their application seems to be a promising direction to follow. Nevertheless, the high complexity of EOs composition and differences in laboratory protocols of the antimicrobial activity assessment hinders the exact estimation of EOs effectiveness. To overcome these disadvantages, in the present work we analysed the effectiveness of volatile and liquid forms of seven EOs (derived from thyme, tea tree, basil, rosemary, eucalyptus, lavender, and menthol mint) against 16 staphylococcal biofilm-forming strains using cohesive set of in vitro techniques, including gas chromatography–mass spectrometry, inverted Petri dish, modified disk-diffusion assay, microdilution techniques, antibiofilm dressing activity measurement, AntiBioVol protocol, fluorescence/confocal microscopy, and dynamic light scattering. Depending on the requirements of the technique, EOs were applied in emulsified or non-emulsified form. The obtained results revealed that application of different in vitro techniques allows us to get a comprehensive set of data and to gain insight into the analysed phenomena. In the course of our investigation, liquid and volatile fractions of thyme EO displayed the highest antibiofilm activity. Liquid fractions of rosemary oil were the second most active against *S. aureus*. Vapour phases of tea tree and lavender oils exhibited the weakest anti-staphylococcal activity. The size of emulsified droplets was the lowest for T-EO and the highest for L-EO. Bearing in mind the limitations of the in vitro study, results from presented analysis may be of pivotal meaning for the potential application of thymol as a antimicrobial agent used to fight against staphylococcal biofilm-based infections.

## 1. Introduction

Biofilm is a cohesive and complex community consisting of microbial cells, embedded within a self-produced matrix that displays protective and nutritional features. The matrix also enables the integrity of biofilm, and in the case of the sessile type of this structure because it facilities adhesion to biotic and abiotic surfaces. Bacteria within the biofilm, compared to their planktonic (non-aggregated) counterparts, demonstrate specific patterns of growth rate, gene transcription, and metabolic activity. It translates into (among others) highly elevated biofilm tolerance/resistance to environmental stress and eradication with antimicrobials [1]. Thus, biofilm is a significant causative factor in a number of persistent, hard-to-heal infections, including these occurring in chronic wounds and bones [2]. Due to biofilm’s persistence, even systemic high-dose antibiotic therapy displays low efficacy; in turn, topical application of antibiotics to treat biofilm-based infections is associated with numerous adverse effects. Therefore, the treatment of biofilm-based, chronic bone and wound infections requires (if possible) surgical intervention and application of antiseptics [3]. As numerous reports indicate microbial resistance to not only antibiotics but also to antiseptics, a growing interest in new antimicrobials and novel ways of their administration is presently observed [4].

Essential oils (EOs) are plant-derived liquids containing numerous compounds of acknowledged and/or alleged antimicrobial activity. Many of the compounds display broad and unspecific mechanisms of action (for example, interaction with the lipids of the cell membrane of microorganism, resulting in metabolic damages and cell death) making them effective against antibiotic-resistant strains and biofilms [5]. The complexity of EOs composition hinders, to some extent, exact understanding of the interplay between their specific components, because various types of interactions, as synergy, antagonism, addition or indifference may occur. On the other hand, this diversity contributes to EOs omnidirectional influence on biofilm (manifested as inhibition of Quorum Sensing (QS), reduction of virulence factors’ expression, or inhibition of biofilm adhesion). Moreover, because various EOs components target diverse sites of microbial cell structure, the application of EOs is not associated with the risk of the development of bacterial resistance [6].

It was reported that the combined use of various EOs modulates bacterial resistance to antibiotics, for example, by targeting efflux pumps, stabilizing molecule form, and by protecting antibiotics against bacterial enzymes [7]. The synergistic action of EOs with antibiotics and antiseptics (“boosting effect”) was also indicated. It was revealed that the application of rosemary, eucalyptus, and thyme oils increases antimicrobial activity of povidone-iodine antiseptic against methicillin-resistant *Staphylococcus aureus* strains up to 136 times [8].

It is worth noting that not only liquid but also volatile forms of EOs display antimicrobial activity. The application of the vapour of EOs provides a high concentration of active compounds to the infection site and limits side effects related to systemic administration and the toxicity being result of direct contact between antimicrobial substances and the issue [9].

Studies on an animal model have confirmed that topical application of EOs promotes the wound healing process. The use of lavender, rosemary, eucalyptus, and basil oils on wounds translates into more favourable results of collagen deposition, closure rate, fibroblasts proliferation, and exudate level [10]. Other research indicates that thyme oil reduces the amount of nitric oxide released in response to burn injuries and facilitates wound healing [11]. The clinical trial has demonstrated the potential of tea tree EO in the therapy of osteomyelitis and wound infection [12]. In turn, 1,8-cineole, a compound found in rosemary and eucalyptus oils, has been reported to act synergistically with amoxicillin and gentamicin in combating MRSA-induced osteomyelitis in rabbits [13]. It should also be noted that such commonly used EOs as St. John’s wort, cinnamon, thyme, rosemary, white poplar, ginger, and notopterygium root, have a beneficial impact on bone features, including mineral turnover normalization, inhibition of bone loss, enhancement of plasma calcium and vitamin D3 level, bone mineral-density improvement, and drop of inflammation and oxidative stress level [14]. Moreover, ylang-ylang, rosemary, eucalyptus, frankincense, tea tree, and wintergreen EOs are able to improve biocompatibility and bone regeneration ability and to prevent microbial colonization [14]. Since EOs are extensively metabolized in the human organism, their bioavailability as a potential systemic agent is limited [15]. However, this obstacle is of rather low meaning in the case of wound treatment, where local effectiveness is primarily required [9]. Taking into consideration the wide range of antimicrobial activity of EOs and their low cytotoxicity, the application of these plant-derived substances as alternatives to antibiotics and antiseptics in the treatment of chronic wound and bone infections is a direction worth to follow and to investigate [16,17,18]. Therefore, the present study aimed to evaluate the antimicrobial and antibiofilm in vitro activity of volatile and liquid fractions of selected EOs against *S. aureus* methicillin-resistant (MRSA) and methicillin-sensitive (MSSA) clinical and reference strains (the key factors of wound and bone infections).

## 2. Results

### 2.1. Assessment of EOs Compositions Using Gas Chromatography Mass Spectrometry

Each EO consists of numerous components; therefore, in the first line of experiment, EOs’ content with regard to presence of antimicrobial substances was analysed using GCMS technique. Thymol and p-cymene were confirmed to be the main components of T-EO. Terpinen-4-ol and γ-terpinene were primarily presented in TT-EO. B-EO was comprised of methyl chavicol and linalool; the main components of R-EO were 1,8-cineole, camphor, and limonene. 1,8-cineole and γ-terpinene predominated in E-EO. M-EO was mainly composed of menthol, menthone, isomenthone, and L-EO of linalyl acetate and linalool. The detailed list of EOs composition is presented in Table S1 in the Supplementary Materials.

### 2.2. Assessment of Biofilm Biomass Level Using Crystal Violet Assay and Biofilm Metabolic Activity Level Using Tetrazolium Chloride Staining

**Figure 1:**
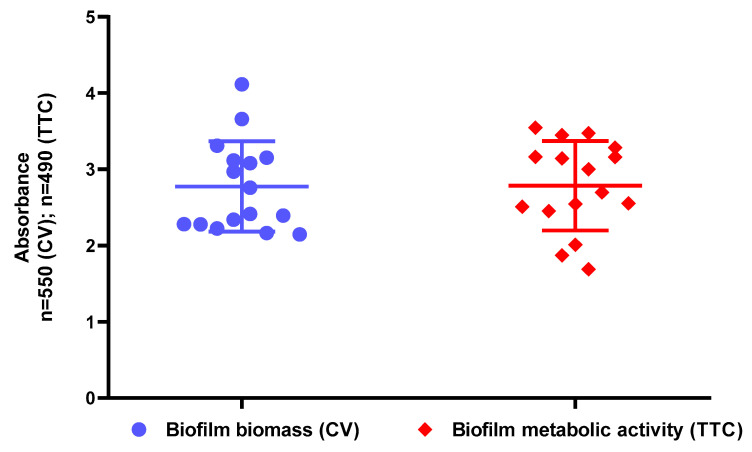
Ability of analysed *S. aureus* strains to form biofilm and assessed with crystal violet (CV) and tetrazolium chloride (TTC) staining.

After confirmation of presence of antimicrobial substances in tested EOs, the ability of all *S. aureus* strains to form biofilm in applied in vitro setting was checked. The results presented in Figure 1 indicate that all staphylococcal strains possess the ability to form in vitro biofilms displaying metabolic activity.

### 2.3. Antimicrobial Activity of All EOs Using Disc Diffusion Method and Inverted Petri Dish Method

**Figure 2:**
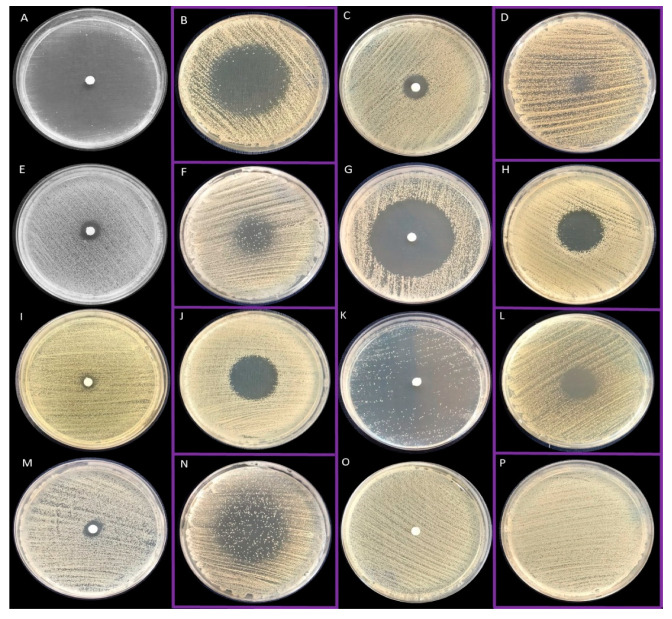
Zones of growth inhibition after the treatment of bacteria with volatile and liquid fractions of EOs assessed with the inverted Petri dish method and disc diffusion technique, respectively. Results of volatile fractions activity are marked with purple frames. (**A**,**B**)—thyme oil (SA 2, SA ATCC 33591, respectively); (**C**,**D**)—tea tree oil (SA 35, SA ATCC 33591, respectively); (**E**,**F**)—basil oil (SA 32, SA 33, respectively); (**G**,**H**)—rosemary oil (SA 33, SA 10, respectively); (**I**,**J**)—eucalyptus oil (SA 5, SA 28, respectively); (**K**,**L**)—menthol mint oil (SA 33, M SA ATCC 33591, respectively); (**M**,**N**)—lavender oil (SA 35, SA 33, respectively); (**O**,**P**)—sodium chloride 0.9% (control setting) (SA 26, SA 27, respectively). The picture (**F**) shows the zone of the partial inhibition of growth.

Next, the antimicrobial activity of EOs’ liquid and volatile fractions was evaluated with standard techniques referred to as the disc diffusion and inverted Petri dish methods, respectively. The representative results from techniques applied are shown in Figure 2. With regard to liquid phases, T-EO and R-EO were the most effective against staphylococcal cells. B-EO, E-EO and L-EO exhibited the lowest antimicrobial activity among tested EOs. In case of specific strains, only zones of partial growth inhibition were observed. The mean diameters of growth inhibition zones being result of exposure of staphylococci to liquid EOs are presented in Table 1. By means of the inverted Petri dish method, volatile fractions of TT-EO, B-EO, and L-EO were characterized as ineffective against the majority of tested strains. Among the investigated EOs, vapours of T-EO displayed the most potent anti-staphylococcal activity. The mean diameters of growth inhibition zones being result of exposure of staphylococci to vapour phases of EOs are presented in Table 2.

### 2.4. Evaluation of the Minimal Inhibitory Concentration (MIC) of Liquid Fractions of All EOs Emulsions in Tween 20 Using Serial Microdilution Method

The aim of this part of the study was to determine the MIC (minimal inhibitory concentration) of liquid fractions of EOs using microdilution method in 96-well plates. Due to the poor solubility of EOs in hydrophilic media such as Tryptic Soy Broth, emulsions in Tween 20 were applied. Firstly, the influence of different Tween 20 concentrations on the growth of planktonic forms of *S. aureus* ATCC 6538 strain was evaluated. The results, presented in Figure S1 in the Supplementary Materials, indicated that addition of up to 1% (*v/v*) Tween 20 did not affect staphylococcal growth. All tested EOs emulsions displayed antimicrobial activity against planktonic forms of analysed clinical and reference *S. aureus* strains. All tested strains were sensitive to EOs emulsions in concentrations equal to or lower than 6.3% (*v/v*). The lowest (the most favourable ones with regard to antimicrobial activity) MIC values were obtained for thyme oil (T-EO) emulsion, while the highest for B-EO and E-EO. Interestingly, clinical MRSA planktonic strains were more susceptible to T-EO emulsions than a reference *S. aureus* ATCC 33591 strain. The MIC values of all EOs emulsions against all strains are presented in Table 3.

### 2.5. Evaluation of the Minimal Biofilm Eradication Concentration (MBEC) and the Minimal Bactericidal Concentration for Biofilm (MBC-B) of Liquid Fractions of All EOs Emulsions in Tween 20

The ability of liquid phases of EOs to eradicate bacterial biofilms was assessed with an MBEC (minimal biofilm eradication concentration) assay. Similar to the MIC assay, dilutions of EOs were performed using Tween 20 as an emulsifier. E-EO and L-EO emulsions exhibited no antibiofilm activity. T-EO emulsion was the most effective among the tested EOs against *S. aureus* biofilms. Minimal biofilm eradication concentrations of T-EO were equal to inhibition values against eight staphylococcal strains. Except for E-EO, liquid fractions of all EOs emulsions exhibited bactericidal activity against individual strains. In the case of nine staphylococcal strains, T-EO emulsions demonstrated bactericidal activity in concentrations equal to MBEC values. The MBEC values of each EO emulsions and all strains are presented in Table 3. The MBC-B (minimal bactericidal concentration for biofilm) values are presented in Table S2 in the Supplementary Materials.

### 2.6. Evaluation of Antibiofilm Activity of All Non-Emulsified EOs’ Liquid Fractions Measured with Modified Antibiofilm Dressing’s Activity Measurement Assay

**Figure 3:**
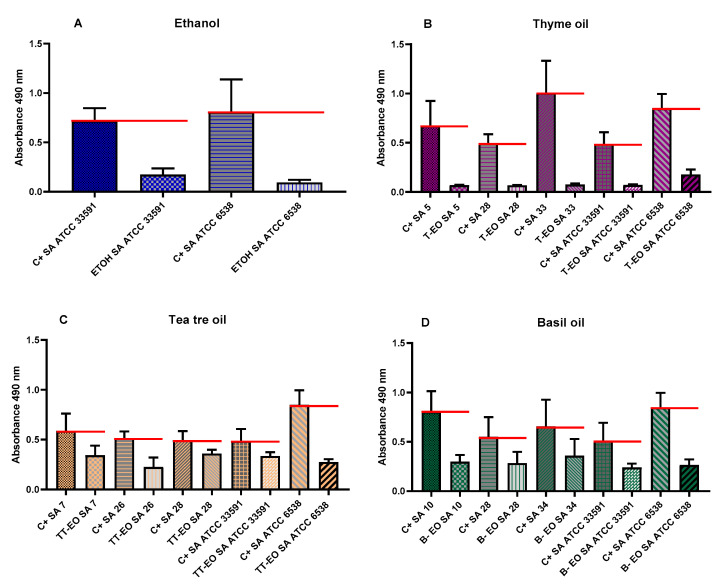

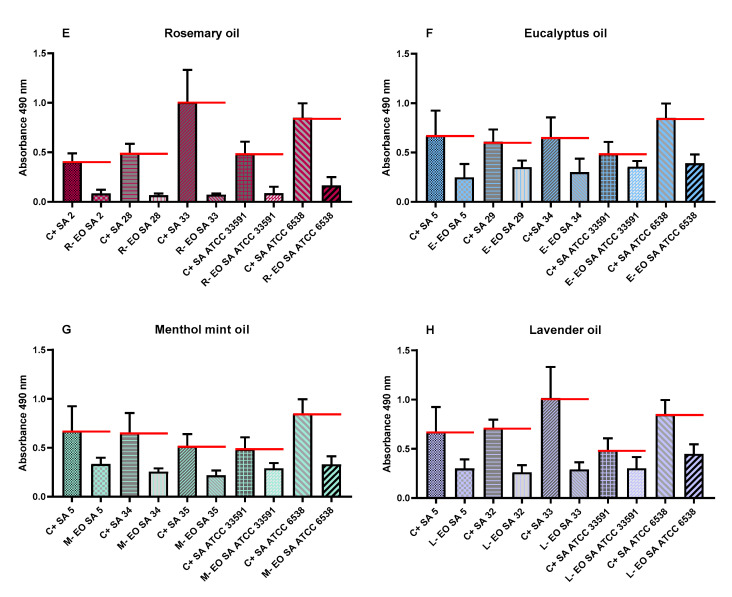
Antibiofilm activity of liquid fractions of non-emulsified EOs and ethanol against *S. aureus* measured with modified A.D.A.M. (antibiofilm dressing’s activity measurement) assay. (**A**–**H**)—results of TTC assay. ETOCH—ethanol, T-EO—thyme oil, TT-EO—tea tree oil, B-EO—basil oil, R-EO—rosemary oil, E-EO—eucalyptus oil, M-EO—menthol mint oil, L-EO—lavender oil, C+ control of growth. Absorbance of growth controls samples are marked with red lines.

The antibiofilm activity of liquid fractions of all non-emulsified and non-diluted EOs against *S. aureus* was determined using a modified A.D.A.M. (antibiofilm dressing’s activity measurement) method. Based on the results of microdilution assays, three different clinical strains for each EO were selected and examined. To provide other research teams with the possibility of performance of this analysis, reference staphylococcal strains were also included. As a substance of proven antimicrobial activity, liquid phases of 96% (*v/v*) ethanol were applied (as controls of test usability). The concentration of EOs released from biocellulose discs was 65.8%. All EOs displayed an ability to eradicate biofilms (from 27% up to 92%). T-EO and R-EO were the most effective against all tested strains. T-EO and R-EO exhibited stronger antibiofilm activity against the *S. aureus* ATCC 33591 strain than ethanol, which served as reference substance. The antibiofilm activity of liquid fractions of non-emulsified EOs and ethanol against selected strains is depicted in Figure 3.

### 2.7. Evaluation of Antibiofilm Activity of All Non-Emulsified Eos’ Volatile Fractions Measured with AntiBioVol Assay

**Figure 4:**
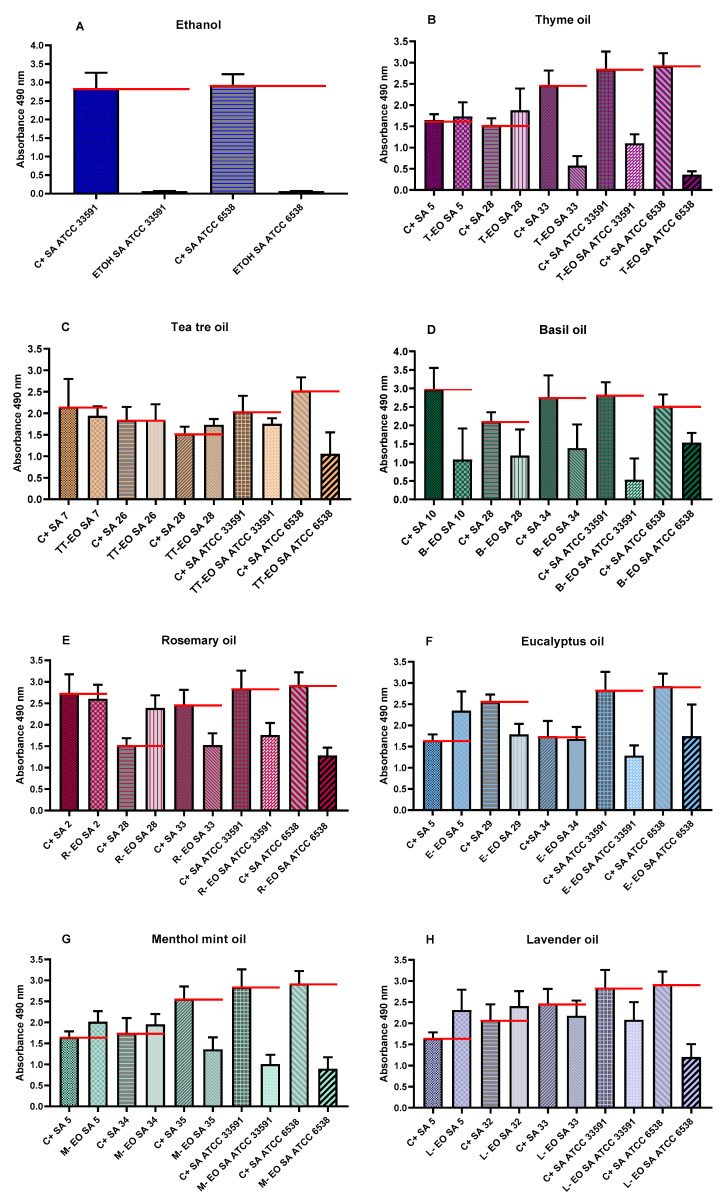

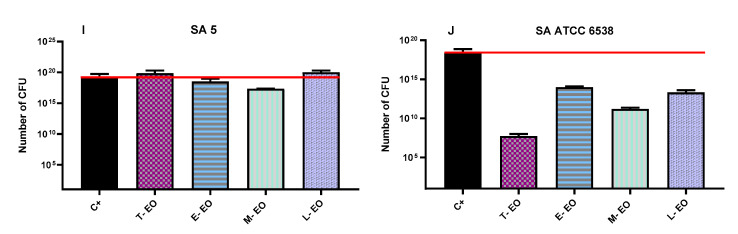
Antibiofilm activity of volatile fractions of non-emulsified EOs and ethanol against *S. aureus* measured with AntiBioVol (antibiofilm activity of volatile compounds) method. (**A**–**H**)—results of TTC assay, (**I**,**J**)—results of quantitative culturing. ETOCH—ethanol, T-EO—thyme oil, TT-EO—tea tree oil, B-EO—basil oil, R-EO—rosemary oil, E-EO—eucalyptus oil, M-EO—menthol mint oil, L-EO—lavender oil, C+ control of growth. Absorbance of growth controls samples are marked with red lines.

Antibiofilm activity of volatile fractions of all non-emulsified and non-diluted EOs against *S. aureus* was determined using AntiBioVol (antibiofilm activity of volatile compounds) method against strains investigated in the modified A.D.A.M. test. As a substance of proven antimicrobial activity, the volatile phase of 96% (*v/v*) ethanol was investigated against reference strains. The differentiated antibiofilm efficacy was observed depending on analysed bacterial strains and the type of oil applied. Volatile fractions of B-EO were the only ones that reduced the number of biofilm cells of all tested strains. In turn, T-EO’s volatile fractions exhibited the strongest antibiofilm activity against reference strains. Growth of SA 5 biofilm was increased after exposure to vapours of all applied EOs (T-EO, E-EO, M-EO, and L-EO). TT-EO and L-EO only slightly eradicated biofilm but in specific cases they enhanced the growth of biofilm formed by clinical strains. In order to determine the influence of volatile fractions of EOs on bacterial cell numbers, quantitative culturing was also performed for two strains exposed to four Eos. SA 5 and SA ATCC 6538 were chosen as the strains in which the opposite impact of EOs was observed in the AntiBioVol assay. The decrease in the number of SA ATCC 6538 cells was observed, while in the case of SA 5, the number of cells was higher or scarcely lower than in control setting. The results of the AntiBioVol (antibiofilm activity of volatile compounds) test are presented in Figure 4.

### 2.8. Visualisation of Impact of T-EO Emulsions’ Liquid Fractions on Staphylococcal Biofilm Using LIVE/DEAD Staining and Epifluorescence Microscopy

**Figure 5:**
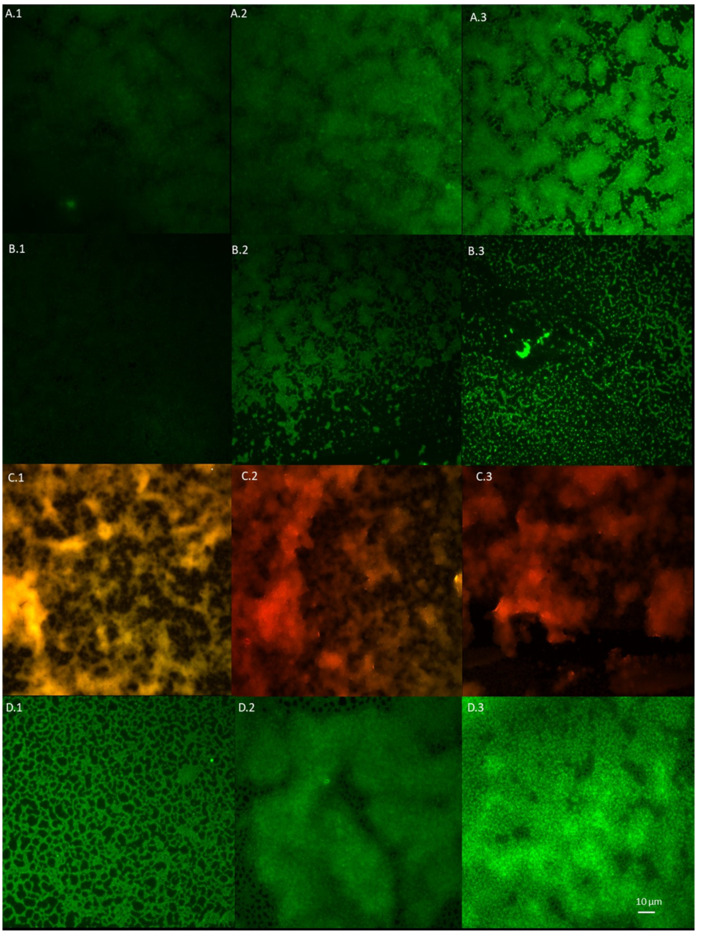
Microphotography of the *S. aureus* ATCC 6538 reference strain biofilm stained with LIVE/DEAD dye. (**A**,**B**)—biofilm exposed to liquid fractions of thyme oil emulsions in concentration 1.6% (*v/v*) (**A.1**–**A.3**) and 0.8% (*v/v*) (**B.1**–**B.3**); (**C.1**–**C.3**)—biofilm treated with 0.1% octenidine and 2% phenoxyethanol solution; (**D.1**–**D.3**)—untreated cells. The red/orange colour shows staphylococcal cells altered/damaged in result of exposure to liquid T-EO emulsion, while green-coloured cells are non-altered, viable cells. Fluorescence microscope Etaluma 600 (magnification 20×).

**Figure 6:**
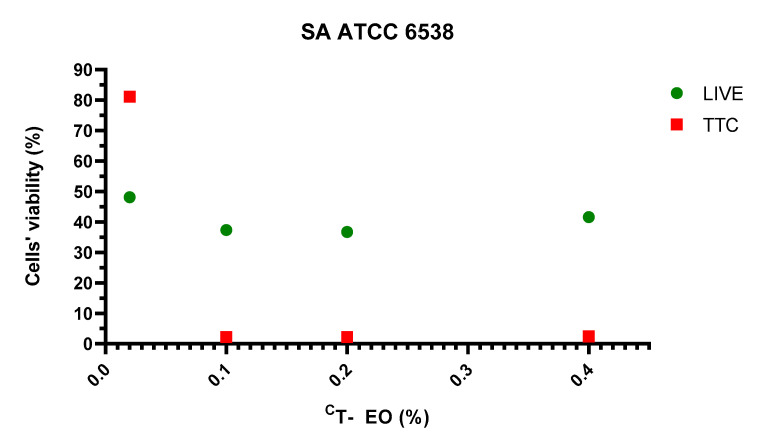
Viability (%) of *S. aureus* ATCC 6538 biofilm treated with liquid fractions of T-EO (thyme oil) emulsions assessed with LIVE (**green colour**) and TTC (**red colour**) dyes.

Based on the above-presented results, T-EO emulsion, as the most potent EO against *S. aureus* biofilms, was chosen for study using fluorescent microscopy. The assessment of antibiofilm activity of the emulsion’s liquid fractions against a reference ATCC 6538 strain, examined with a LIVE/DEAD dye, indicated 57% biofilm cell reduction in concentration 0.4% (*v/v*) (concentration equal to MBEC value evaluated with a TTC indicator). Visualisation of the strain ATCC 6538 biofilm stained with LIVE/DEAD dye is presented in Figure 5. Graphical comparison of ATCC 6538 biofilm viability treated with liquid phases of T-EO emulsion evaluated with LIVE/DEAD and TTC dyes is presented in Figure 6. An interesting phenomenon manifested by significant drop of metabolic activity occurred after exposure to T-EO concentration >0.1% (*v/v*) with maintaining level of staphylococcal cell integrity (at level of ~35%) was observed.

### 2.9. Three Dimensional Visualization of Alterations of Staphylococcal Biofilm Exposed to Volatile Fractions of R-EO

**Figure 7:**
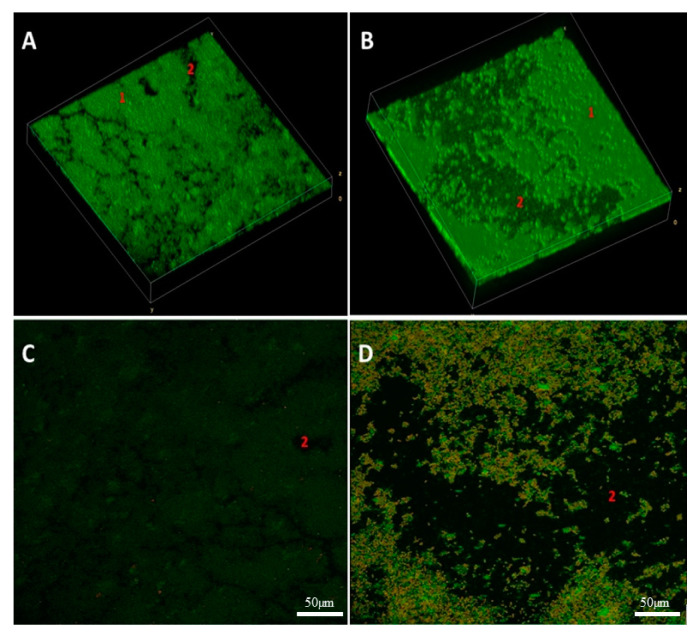
Impact of vapour phase of R-EO on *S. aureus* ATCC 6538 biofilm. (**A**,**B**)—volumetric data showing untreated and treated biofilm, respectively. (**1**)—non-altered fragment of biofilms; (**2**)—loss of biofilm volume. (**C**,**D**)—staphylococcal biofilm cells treated and untreated with R-EO, respectively. The red/orange colour shows staphylococcal cells altered/damaged in result of exposure to vapour R-EO, while green-coloured cells are non-altered, viable cells. Moreover, the more dark (less green) picture is, the less live cells are captured in this particular field of vision.

The impact of vapour R-EO on *S. aureus* ATCC 6538 biofilm was analysed using 3D confocal microscopy and parametric processing of visual data obtained (Figure 7). Volumetric images revealed high reduction of staphylococcal biofilm after exposure to R-EO (manifested in the form of loss of biofilm (Figure 7B, fragments pointed with number “2”) and higher share of damaged/compromised cells (Figure 7D) compared to the staphylococcal biofilm untreated with R-EO (Figure 7A,C).

### 2.10. Size of Emulsified EOs Droplets

Finally, because the correlation between EO emulsion droplet size was recently shown to be another factor of impact on antimicrobial activity, the average diameters of droplets of emulsified EOs were measured. The results (in increasing order of diameter) were as follows: T-EO: 637 ± 287 nm; R-EO: 783 ± 69 nm; TT-EO: 1079 ± 59 nm; M-EO: 1515 ± 116 nm; B-EO: 2172 ± 813 nm; E-EO:2201 ± 110 nm; L-EO: 3531 ± 204 nm.

## 3. Discussion

The results of numerous studies indicate that EOs are a promising alternative to antibiotics thanks to their broad spectrum of antimicrobial activity and unspecific mode of action, which correlates with low risk of microbial resistance emergence [19,20]. As highly lipophilic substances, EOs bind to and disrupt the integrity of microbial cell walls and membrane structures, resulting in cell lysis [21]. It is reported that EOs may also exhibit such other mechanisms of action against biofilms as blocking the quorum-sense system, inhibiting the transcription of flagellar genes, interfering with bacterial motility, reducing the bacterial adherence to inert surfaces, increasing the oxidative stress in microbial cells, and blocking the productions of enzymes [22].

Essential oils are characterized by complex and variable composition, high volatility, and poor water solubility [23]. There are many factors which have impacts on the molecular composition of EOs, including seasonal climatic variations, intraspecies variability, and the method of oil extraction [24]. It has been found that two or three major classes of substances (and their concentrations) determine EOs biological activity to the major extent [23]. EOs contain a high level of phenolic compounds, e.g., carvacrol, eugenol, and thymol, which are substances with a proven, strong antibacterial effect [25]. Therefore, in the first part of the study we have evaluated the composition of each EO and confirmed the presence of compounds of antimicrobial activity (Table S1). It is noteworthy that vapour forms of EOs are reported to have higher antimicrobial effect than the liquid fractions [26,27,28,29]. It is suggested that lipophilic molecules in the EOs’ aqueous phase associate, form micelles, and restrain the attachment of EOs to microorganisms. The vapour phase is devoid of this disadvantage that allows antimicrobials to be easily released and to strongly attach to microbial structures [30]. Due to the aforementioned volatility and water immiscibility of EOs, evaluation of their antimicrobial activity using in vitro assays displays certain limitations. Therefore, we have analysed antibacterial effectiveness of EOs’ both fractions and have compared results using differentiated methodological approaches. In the present study, the antimicrobial activity of seven essential oils has been investigated against fourteen clinical and two reference *S. aureus* strains. The strains forming biofilm in the most robust manner have been selected (Figure 1) for further analyses. First, the evaluation of antimicrobial activity of liquid and vapour fractions of EOs was performed using disc diffusion method and inverted Petri dish assay, respectively. Results of the inverted Petri dish assay demonstrated that the strongest antimicrobial activity was displayed by the liquid phases of T-EO and R-EO; a moderate effect was shown for TT-EO, M-EO; and the weakest effect for B-EO, E-EO and L-EO (Figure 2, Table 1). In turn, Chao et al., analysing antibacterial effect of EOs against a MRSA reference strain, indicated the following (in decreasing order) activity of liquid fractions of these substances: T-EO > TT-EO > R-EO = L-EO > B-EO > E-EO [31]. The discrepancies of results presented by Chao et al. with outcomes presented in this work (concerning difference in activity of R-EO but not T-EO) may be caused by the different volume of used EOs, resulted in various level of diffusion of these substances through the agar medium. It is noteworthy that other studies have also confirmed the significant antimicrobial activity of T-EO liquids against clinical and reference MRSA and MSSA strains [32,33,34,35], similar to the results presented in this study. The zones of growth inhibition of reference and clinical staphylococcal strains after exposure to T-EO liquid fractions, presented in work of Tohidpour et al., where the disc diffusion method was applied, ranged from 12 to 35 mm, while Kryvtsova et al., using a well diffusion assay, observed formation of zones of 45–66 mm diameter [36,37]. Contrary to these results and results shown in this work, Mardafkan et al. have presented no activity of T-EO liquid phases against *S. aureus* [38]. The different thymol content—the main T-EO’s compound responsible for antimicrobial activity—may be the reason standing behind the observed differences in outcomes. T-EO, applied in the research of Mardafkan et al., contained thymol in concentrations of 30% and benzene in concentrations of 14%, whereas T-EO applied in our study comprised a higher concentration of thymol (44%) and p-cymene (27%) but not benzene (Table S1). Lemos et al. have shown that T-EO where the thymol concentration was equal to 53% exerted an MIC against *S. aureus* equal to 0.02 mg/mL, while the 40% concentration of thymol, corresponded with an MIC value of 0.17 mg/mL [39].

Similarly, the results obtained for liquid phases of EOs when vapour phases were also analysed showed that the greatest zones of microbial growth inhibition were detected for T-EO. No zones of growth inhibition were demonstrated for B-EO and L-EO (Figure 2, Table 2). These results cannot be directly compared with results from other studies due to diversity of Inverted Petri Dish assay protocols applied. Nevertheless, the anti-staphylococcal efficacy of vapour phases of thyme oil has been proven in numerous studies [40,41,42,43,44,45,46,47]. On the other hand, some studies have also indicated significant activity of TT-EO, E-EO, B-EO, L-EO vapour against *S. aureus* [40,42,47,48,49], whereas other studies are consistent with our results [40,41]. Differences in applied methodologies such as volume and concentration of oil used, addition of oil solvents, testing several samples on the same plate, paper disc diameter, or agar surface height, are suggested to explain the discrepancies in outcomes. As it has been reported in our previous study, all these factors may have a significant impact on the diameter of the obtained zone of growth inhibition [50]. The above conclusion is also strongly upheld by the data provided by Aber et al. who showed dose-dependent antibacterial activity of essential oils’ vapour fractions [51].

In the subsequent step of our investigation, we have evaluated antibacterial effect of liquid fractions of EO emulsions on planktonic and biofilm cells of *S. aureus* using standard microdilution methods. Tween 20 was used as non- ionic surfactant to enhance solubilization and reduce evaporation of EOs [52]. According to our previous research, addition of 0.5% (*v/v*) Tween 20 improved anti-staphylococcal activity of TT-EO liquid fractions [53]. As it is presented in Figure S1 in Supplementary Materials, Tween 20 did not inhibit growth of *S. aureus* planktonic cells in the concentrations used for emulsion preparation. The liquid fractions of all tested EOs emulsions effectively inhibited growth of *S. aureus* planktonic forms (Table 3). The lowest (most favourable outcome) MIC—minimal inhibitory concentration—values were obtained for T-EO and M-EO emulsions. Numerous studies have confirmed our results [54,55,56]. In case of antibiofilm activity of EOs liquid phases, T-EO and TT-EO emulsions were the most potent ones. B-EO, R-EO, and M-EO emulsions eradicated biofilms of particular strains, whereas E-EO and L-EO emulsions were inactive (Table 3). Except for T-EO, MBEC (minimal biofilm eradication concentration) values of EOs were higher than MIC. Such results stay in line with generally accepted fact of protective function of biofilm matrix resulting, among others, in increased tolerance of cells on antimicrobial substances [57]. Oussalah et al. and Horváth et al. have reported the same MIC values against reference *S. aureus* strain as these presented in this research for T-EO and M-EO emulsions, respectively [54,58]. It is noteworthy that the major components and their concentrations in T-EO applied by these research teams were comparable (Table S1). Similarly, in our previous research, the same MIC and MBEC values of non-emulsified T-EO have been recorded against *S. aureus* reference strain. Considering the above, it is suggested that an emulsifier, if applied in an appropriate concentration, does not increase the oil’s liquid fraction antimicrobial activity. As mentioned before, thymol is thought to be the compound accountable for T-EO properties. Li et al. have indicated that, in case of thymol, surfactant addition may even reduce its antimicrobial activity [59]. One of proposed explanations of this phenomenon is the trapping thymol by the surfactant at the micelle oil-water interface. This phenomenon reduces the soluble thymol content in the aqueous phase. However, low Tween 20 concentration in our samples may explain why reduction of activity was not observed. It has been reported that thymol’s activity depends on the bacterial species that is applied against and physical properties of the molecule. The presence of the hydroxyl group and a system of delocalized electrons plays an important role in the antimicrobial activity of thymol (and its isomer—carvacrol), by disturbing bacterial membrane functions, altering lipid barrier, depleting ATP, and finally, causing bacterial cell dead, [60]. This complex mechanism may be responsible for observed antibiofilm effect of T-EO emulsion against several *S. aureus*, when applied concentrations of T-EO were equal of these defined as minimal inhibitory ones (Table 3, Table S2). Gömöri et al. have demonstrated that minimal bactericidal concentration (MBC) of T-EO against MRSA and MSSA reference strains were only 2-fold higher than MIC values [61]. In our study, liquid fractions of TT-EO emulsions exerted antibiofilm effect in concentration ranges from 6.3 to 0.8% (*v/v*) (Table 3). These results are consistent with results of our previous studies in which we observed that liquid fractions of the EOs are able to reduce viability of staphylococcal biofilm cells in microdilution assays [50,53]. Feng et al. have indicated complete removal of mature MRSA biofilm after the exposure to 0.32% TT-EO [62]. In turn, MBEC and MBC-B values of liquid fractions of emulsified R-EO, M-EO, and B-EO differed with regard to staphylococcal strains they were applied against (Table 3). Analysing influence of R-EO on biofilm of clinical MSSA and MRSA clinical and reference strains, other researchers revealed analogical trends to these shown in this work [8,63]. Nazir et al. have found that B-EO in 5% (*v/v*) concentration inhibited 20% staphylococcal biofilm cells, whereas concentrated oil was able to inhibit 55% of these cells [64]. No MBEC value was also obtained when oil concentration of 50 μL/mL was applied [65]. Kifer et al. have found that MBEC value of menthol, a main component of M-EO, ranged from 3.21 to 6.35 mg/mL against MRSA and MSSA clinical and reference strains [66]. In the same study MBEC values of the component predominating in E-EO (1,8-cineole) were in the concentration range from 128 to 254 mg/mL [66]. These data stay in line with results from our study in which we showed that M-EO emulsions liquid fractions display higher antibiofilm activity than E-EO (Table 3). On the other hand, Merghni et al. have demonstrated staphylococcal biofilm reduction above 80% after the treatment with liquid phases of 0.2 mg/mL E-EO as well as 0.2 mg/mL 1,8-cineole [67]. Liquid phases of L-EO emulsions tested in this research effectively eradicated only biofilm of a *S. aureus* ATCC 6538 strain at minimal concentration equal to 1.6% (*v/v*) (Table 3). Research provided by other teams have indicated a 3-fold higher MBEC value against a MRSA reference strain and MBEC equal to 12.5 μL/mL against the susceptible ones [65,68]. The low reduction of biofilms formed by clinical MRSA and reference MSSA strains was demonstrated after the exposure to liquid fractions of L-EO, linalyl acetate, and linalool [69,70]. To gain broader insight into phenomena analysed, we have performed tests of antibiofilm activity of non-emulsified liquid and volatile fractions of EOs against selected strains of *S. aureus* using another set of methodological settings. We have applied that recently developed and modified A.D.A.M. (antibiofilm dressing’s activity measurement) assay and AntiBioVol (antibiofilm activity of volatile compounds) methodology for the assessment of liquid and volatile fractions, respectively. Both models use agar as a surface for biofilm culturing and provide semi-quantitative type of data. According to the results received in the modified A.D.A.M. assay, liquid fractions of all essential oils possessed the ability to reduce viability of staphylococcal biofilms. The T-EO and R-EO were the most effective ones (Figure 3). Antibiofilm activity of non- emulsified T-EO and E-EO liquid fractions was assessed in our previous study with a similar methodology, though the oil-soaked dressing was applied directly on biofilm cells [71]. The previous study also demonstrated about 60% and 50% reduction of biofilm cells viability for T-EO and E-EO, respectively [71]. The outcomes of AntiBioVol assay have revealed that only volatile fractions of B-EO reduced viability of biofilms of all tested strains (Figure 4). Antibiofilm activity of vapour of other EOs was strain-dependent. Interestingly, volatile fractions of all EOs possessed the ability to decrease level of metabolically active biofilm cells of SA ATCC 6538 strain (and cell number in case of four oils), whereas the level of metabolic viability and cell number of SA 5 biofilm cells increased after exposure to each tested oil (Figure 4). As the analyses were performed in high number of repeats and displayed high cohesion, this specific result additionally underlines inter-species variability in answer of staphylococcal strains to exposure to specific EOs. It also shows importance of using the appropriate number of strains for evaluation of EOs activity to not omit such important phenomena. Finally, the impact of liquid fractions of T-EO emulsion on SA ATCC 6538 biofilm cells viability and membrane integrity was evaluated (Figure 5 and Figure 6). The significant drop of metabolic activity occurred after exposure to T-EO concentration >0.1% (*v/v*) with maintaining level of staphylococcal cell integrity (at level of ~35%) was observed. Considering the fact that liquid fractions of T-EO emulsion demonstrated bactericidal activity in concentration 0.4% (*v/v*) (Table S2), it may be assumed that the oil affects bacterial cells not only by targeting membrane but also via other mechanisms of more bacteriostatic nature. It was recently proposed that thymol may exert antibiofilm activity by inhibiting virulence factors such as PIA and hemolysin synthesis [72] and to act in similar manner as its isomer, carvacrol, by affecting genes coding for quorum sensing process [60].

In the present study, the broad spectrum of methods for the assessment of EOs’ antimicrobial activity was applied. In case of non-biofilm forms of microbial communities, the significantly lower activity of liquid fractions of TT-EO, B-EO, E-EO, M-EO, and L-EO has been demonstrated using disc diffusion method comparing to the MIC assay. The discrepancies may result from the fact that usability of disc diffusion technique for such lipophilic substances as essential oils is limited. Furthermore, application of the emulsifier in MIC assessment may have improved the efficacy of EOs components.

No antibiofilm activity of E-EO and L-EO emulsions’ liquid fractions has been indicated with use of microdilution method, whereas reduction of biofilm cells’ viability after exposure to these non- emulsified EOs was demonstrated using modified A.D.A.M. methodology. It should be stressed that different surfaces for biofilm formation (polystyrene in MBEC assay and agar in the modified A.D.A.M. test) may influence biofilm adhesion, density and contribute to level of EOs’ effectiveness. Moreover, in MBEC assay EOs emulsions are in constant contact with bacterial cells, while in A.D.A.M. assay, EOs are gradually released from biocellulose discs to the medium in which bacteria are immersed in. In turn, rate of EOs components release from biocellulose may have an effect on eradication activity of particular EOs.

There were no zones of growth inhibition obtained with inverted Petri dish method after the exposure of *S. aureus* cells to B-EO, though significant reduction of biofilm cells viability after exposure to this EO was measured with AntiBioVol test. More potent activity of B-EO’s vapour fractions against mature biofilm than against microbial planktonic cells suggests that activity of this oil may affect various stages of biofilm life-cycle. As it was mentioned, numerous factors have an impact on inverted Petri dish method’s accuracy. Furthermore, in AntiBioVol technique EOs are applied directly under the entire biofilm surface, while in inverted Petri dish EOs vapour are spread over the 90-mm Petri dish. Different volumes of EOs used in both assays are also of high impact for obtained results.

The strongest antimicrobial and antibiofilm activity were determined for thyme oil. Rosemary and menthol mint oils also displayed significant anti-staphylococcal activity. The main components of the oils (thymol in thyme oil, 1,8-cineole in rosemary oil, and menthol in menthol mint oil) are, to a major extent, accountable for their activity. Studies indicated that the antimicrobial effect of monoterpenes such as (+)-menthol and thymol is partially observed due to the disruption of the lipid fraction of the plasma membrane, causing a changed permeability and leakage of intracellular materials [73]. The research of Li et al. [74] showed that 1,8-cineole changed the shape and size of the bacterial cell (for both Gram-negative and Gram-positive bacteria). In addition, bacterial cells treated with this compound underwent apoptosis, because they showed a strong condensation of nuclear chromatin located in the central part of the nucleoplasm [74]. 1,8-cineole is the component also presented in eucalyptus oil; it may be assumed that better antimicrobial activity of rosemary oil is the result of synergistic action of eucalyptol and other compounds of the oil, as was suggested by Bajalan et al. [75]

In this work, we applied a broad spectrum of analytical techniques to assess the impact of EOs on staphylococcal biofilm. The reason behind this agenda was the fact that there are numerous variables related to this seemingly easy-to-perform research, including variances in EOs’ composition, intraspecies variability, and crucial differences in methodological approaches (related to fraction of EO tested, type of surface used for growth, and the various volumes of EOs used, to name just the most important ones). It should be mentioned that such an approach is increasingly being applied, especially in the studies on the impact of various antimicrobials against biofilms [76]. Therefore, we hypothesized that application of prolific in vitro techniques allows us to overcome these challenges and indicates that antibiofilm activity of specific (or some) EOs prevail over others when the abovementioned specific methodologies are applied as a whole. Indeed, the data obtained in this study indicates T-EO (consisting mostly of thymol) as the most potent one. It is noteworthy that Multu-Inglok et al. [77] presented the reverse correlation between the size of emulsified EO and its antimicrobial activity (the smaller the size of droplets, the higher the antimicrobial potential). In our study, the emulsified T-EO droplets were of the lowest, 637 nm diameter, while the size of emulsified L-EO droplets (which displayed low antimicrobial activity in majority of tests) were over 5 times bigger (3531 nm), confirming data presented by above-mentioned research team. It explicitly shows that the number of variables that should be taken under consideration during analysis of interactions of EOs with microorganisms (in biofilm form, especially) may be even higher than previously assumed. Nevertheless, bearing in mind all limitations of the in vitro study, we believe that this conclusion may be of pivotal meaning in subsequent lines of investigation and potential application of thymol in the character of new, antimicrobial agent used to fight against staphylococcal biofilm-based infections.

## 4. Materials and Methods

### 4.1. Microorganisms and Culture Conditions

Two reference strains, *Staphylococcus aureus* 6538 and 33591 from the American Type and Culture Collection (ATCC) and fourteen clinical isolates from bone (7 strains) and wound infections (7 strains) were analysed in this study. Three strains from each group were MSSA strains (methicillin-susceptible *Staphylococcus aureus*), and four were MRSA strains (methicillin-resistant *Staphylococcus aureus*). The list of the strains is presented in Table 4. The strains are part of the Strain and Line Collection of Pharmaceutical Microbiology and Parasitology Department of the Medical University of Wroclaw. All clinical strains applied in this study are part of collection of strains of Department of Pharmaceutical Microbiology and Parasitology of Wroclaw Medical University. The strains were obtained in year 2016 during the internal Wroclaw Medical University SUB. D198.16.001 project: “The insight into biofilm-related properties of clinical microorganisms and possibilities of their eradication”. All patients provided a written consent to participate in the trial and allowed the material obtained during the study (exudate, bioptates, microorganisms) to be used for scientific purposes. The study was approved by the Bioethical Committee of Wroclaw Medical University, protocol # 8/2016.

All strains were cultured overnight before the experiments at 37 °C in Tryptic Soy Broth medium (TSB, Biomaxima, Lublin, Poland). Subsequently, 0.5 McFarland suspensions were established in saline (NaCl, Stanlab, Lublin, Poland) using a densitometer (Densilameter II Erba Lachema, Brno, the Czech Republic) and applied in each test.

### 4.2. Essential Oils

In this study, the antimicrobial activity of volatile and liquid fractions of EOs was investigated. Due to the volatility of EOs, individual essential oils and control settings were examined on separate plates.

The following seven commercial EOs were examined in this study:

- Thyme oil (T-EO, obtained from *Thymus vulgaris* L. herb) was purchased from Etja, Elblag, Poland;
- Tea tree oil (TT-EO, obtained from *Melaleuca alternifolia* Cheel. leaves) was purchased from Pharmatech, Zukowo, Poland;
- Basil oil (B-EO, obtained from *Ocimum basilicum* L. leaves and flowers) was purchased from Nanga, Zlotow, Poland;
- Rosemary oil (R-EO, obtained from *Rosmarinus officinalis* L. flowering shoots) was purchased from Nanga, Zlotow, Poland;
- Eucalyptus oil (E-EO, *obtained from Eucalyptus globulus* Labill. leaves and twigs) was purchased from Pharmatech, Zukowo, Poland;
- Lavender oil (L-EO, obtained from *Lavandula angustifolia* Mill. flowering herb) was purchased from Kej, Cirkowice, Poland;
- Menthol mint oil (M-EO, obtained from *Mentha arvensis* L. leaves) was purchased from Optima Natura, Grodki, Poland.

### 4.3. GC-MS (Gas Chromatography Mass Spectrometry) Analysis of the Tested EOs Composition

#### 4.3.1. Essential Oil Preparation

Essential oils (EO) were diluted with hexane (JTB, GB), vortexed, and immediately analysed. All analyses were performed in triplicate.

#### 4.3.2. GC-MS Analysis

Analysis was performed using Agilent 7890B GC system coupled with 7000GC/TQ system connected to PAL RSI85 autosampler (Agilent Technologies, Palo Alto, CA, USA). The column used was HP-5 MS; 30 m × 0.25 mm × 0.25 µm (J&W, Agilent Technologies, Palo Alto, CA, USA) with helium used as a carrier gas at the total flow of 1 mL/min. Chromatographic conditions were as follows: split injection in a ratio 100:1, the injector was set on 250 °C, oven temperature program was: 50 °C held for 1 min, then 4 °C/min up to 130 °C, 10 °C/min to 280 °C, and then isothermal for 2 min. The MS detector operated in the electronic impact ionization mode at 70 eV, transfer line, source, and quadrupole temperatures were set at 320, 230, and 150 °C, respectively. Masses were registered in a range from 30 to 400 *m/z*. Peaks were identified in MassHunter Workstation Software Version B.08.00 coupled with the NIST17 mass spectra library and accomplished by comparison with linear retention indexes. The relative abundance of each EO constituent was expressed as percentage content based on the peak area normalization. Due to the obtained results, none of the analysed EOs is of pharmacopeial grade. However, the analysis was not performed in accordance with normalization procedure from Polish Pharmacopea XI (different column and different temperature program).

### 4.4. Assessment of Biofilm Biomass Level Using Crystal Violet Assay

To assess total biofilm mass, crystal violet staining was applied. In the first step, 100 µL of all 0.5 MF bacterial suspensions diluted 1000 times in TSB (Tryptic Soy Broth, Biomaxima, Lublin, Poland) medium were added to the wells of 96-well plates (Wuxi Nest Biotechnology, Wuxi, China) and incubated under static conditions for 24 h at 37 °C. After the supernatant fluid was removed, the plate was dried for 10 min (37 °C), and the biofilm was dyed with 100 µL of 20% (*v/v*) crystal violet solution (Aqua-med, Lodz, Poland). The plate was kept at RT for 10 min, biofilm cells were washed twice with 100 µL of saline, and the plate was returned to the incubator (37 °C) for 10 min once more. Subsequently, 100 µL of 30% (*v/v*) acetic acid (Chempur, Piekary Slaskie, Poland) solution was poured into the wells, and the plate was stirred for 30 min at 350 rpm shaker (Mini-shaker PSU-2T, Biosan SIA, Riga, Latvia). The plate’s content was transferred to a fresh 96-well plate, and absorbance was measured at 550 nm using a spectrophotometer (Multiskan Go, Thermo Fisher Scientific, Vantaa, Finland). The experiment was performed twice with six replicates.

### 4.5. Assessment of Biofilm Activity Level Using TTC Staining

Biofilm culturing was performed as described in the crystal violet assay. Subsequently, tetrazolium chloride assay was carried out as follows: 100 µL of 0.1% (*w/v*) TTC solution (2,3,5-triphenyl-tetrazolium chloride, AppliChem Gmbh, Damstadt, Germany) in TSB (Tryptic Soy Broth, Biomaxima, Lublin, Poland) was added to the cells for 2 h (37 °C). The suspension was aspirated, the plate was dried (10 min/37 °C), and 100 µL of methanol was added (Stanlab, Lublin, Poland). The plate was shaken (30 min at 350 rpm), and the solution was carried to the fresh wells of a 96-well plate. Absorbance was measured at 490 nm. The experiment was performed twice with six replicates.

### 4.6. Evaluation of the Antimicrobial Activity of All EOs Using Disc Diffusion Method and Inverted Petri Dish Method

The experiments were performed using Mueller–Hinton agar (Biomaxima, Lublin, Poland) plates (90 mm diameter, 14.2 mm height, Noex, Komorniki, Poland). The agar layer was 5 mm thick. Standard paper discs (diameter of 6 mm, 0.5 mm of thickness) were placed in a 48-well plate (Thermo Fisher Scientific, Waltham, MA, USA), soaked with 0.2 mL of each EOs or saline (as control of bacterial growth) (NaCl, Stanlab, Lublin, Poland), wrapped with tape and kept refrigerated for 30 min. The abovementioned suspensions of all strains (two references and fourteen clinical) (0.5 McFarland) were cultured onto the plates. To evaluate the antimicrobial activity of liquid fractions of all EOs, the paper discs were placed onto the agar, while to assess the activity of vapour fractions onto the plate lids. The plates were sealed with parafilm and incubated for 24 h at 37 °C. Microbial growth inhibition zones were measured (in mm) afterwards using a ruler. Zones of partial growth inhibition were evaluated (in mm) if no total inhibition was observed. In the case of unequal zones, a shorter diameter was included. Each experimental setting was performed in triplicate, and the mean diameter was calculated.

### 4.7. Evaluation of Minimal Inhibitory and Minimal Biofilm Eradication Concentrations of Liquid Fractions of EOs Emulsions

The minimal inhibitory concentration (MIC) and minimal biofilm eradication concentration (MBEC) were assessed for liquid fractions of all EOs against each tested strain. There were three replicates performed in two separate repetitions.

Due to the poor solubility of EOs in a water medium, Tween 20 (Zielony Klub, Kielce, Poland) was used as an emulsifier. Each dilution of the EOs was prepared in a separate 15 mL falcon tube (Flmedica, Padova, Italy). First, 2-fold dilution of EO was performed in Tryptic Soy Broth (TSB, Biomaxima, Lublin, Poland) with 1% (*v/v*) Tween 20 and vortexed (Micro-shaker type 326 m, Premed, Marki, Poland) for 30 min/RT. Other geometric dilutions were prepared in TSB and vortexed for 30 s. For the minimal inhibitory concentration (MIC) purpose, the 0.5 MacFarland suspensions of all strains were diluted 1000× in TSB, and 100 µL were added to 96-well plates (Jet Bio-Filtration Co. Ltd., Guanzhou, China). Subsequently, 100 µL of EOs emulsions were poured, and the plates were incubated for 24 h at 37 °C with continuous shaking at 350 rpm (Mini-shaker PSU-2T, Biosan SIA, Riga, Latvia). After incubation, 20 µL of 1% (*w/v*) TTC (2,3,5-triphenyl-tetrazolium chloride, AppliChem Gmbh, Damstadt, Germany) solution in TSB was added, incubation has proceeded for 2 h under the same conditions. Absorbance was measured at wavelength *λ* = 580 nm using a spectrophotometer (Multiskan Go, Thermo Fisher Scientific, Vantaa, Finland) before and right after the incubation with EOs. In the MBEC assay (minimal biofilm eradication concentration), the microorganisms’ suspensions were prepared likewise, and 100 µL were poured into the wells of 96-well plates and 100 µL of TSB was added. The plates were incubated for 24 h/37 °C to form biofilm. Next, the medium was replaced with 200 µL of EOs emulsions (in the concentration range 50–0.02% (*v/v*)), and the plates were incubated again for 24 h at 37 °C. EOs emulsions were removed, 200 µL of 0.1% (*w/v*) TTC solution in TSB was poured into the biofilm wells, and the plates were incubated for 2 h at 37 °C. The medium was replaced with the same volume of methanol (Stanlab, Lublin, Poland) and glacial acetic acid solution (Chempur, Piekary Slaskie, Poland) (in a ratio of 9:1), and the plates were shaken at 350 rpm for 30 min at RT. A total of 100 µL was transferred to fresh 96-well plates and absorbance was measured at 490 nm wavelength. The MIC and MBEC (%) (*v/v*) values were assessed as the first well where no colour was observed after incubation with TTC. In both assays, controls of microorganisms’ growth and controls of medium sterility were applied. Moreover, the antimicrobial activity of Tween 20 (in the concentration range 1–0.002% (*v/v*)) against *S. aureus* 6538 planktonic forms was investigated.

Additionally, the content of the wells corresponding to MBEC values was transferred to glass tubes with 5 mL of TSB medium and incubated overnight at 37 °C. The MBC-B (minimal bactericidal concentration for biofilm) values were assessed in the tubes where no visible growth was observed.

Based on the MIC and MBEC results, three clinical strains the most susceptible to the liquid fractions of EOs and two reference *S. aureus* strains were selected for further antimicrobial experiments.

### 4.8. Evaluation of Antibiofilm Activity of All Non-Emulsified EOs’ Liquid Fractions Using Modified A.D.A.M. (Antibiofilm Dressing’s Activity Measurement) Assay

The assay was a modification of the protocol presented in our previous study [78]. The following steps of the experiment were performed:

#### 4.8.1. Biofilm Plugs Preparation

Brain Heart Infusion Broth (BHI, Biomaxima, Lublin, Poland) and 2% (*w/v*) of Bacteriological Lab Agar (Biomaxima, Lublin, Poland) were used for filling the wells of a 24-well plate (further referred to as Plate 1) (Wuxi Nest Biotechnology, Wuxi, China) to half their height. The plate was left for agar solidification. Subsequently, agar plugs 8 mm in diameter were cut out of each well using a cork-borer. The plugs were divided into two equal parts. One part of the divided plugs was discarded. The second part was placed in a new 24-well plate (later referred to as Plate 2). The selected strains’ suspensions, prepared as described in the Section 4.1, were then diluted one thousand times in Tryptic Soy Broth medium (TSB, Biomaxima, Lublin, Poland), and 2 mL was added to the plugs—containing wells of Plate 2 and incubated at 37 °C for 24 h. During the incubation, biofilm was formed at the top of the plugs.

#### 4.8.2. Treatment with EOs

To assess the antibiofilm activity of all tested EOs, biocellulose dressings were prepared as follows:

A *Komagataeibacter xylinus* ATCC 53524 strain was cultured stativity in Herstin–Schramm (H-S) medium for 7 days at 28 °C for cellulose production. The medium was composed of 2% (*w/v*) glucose (Chempur, Piekary Slaskie, Poland), 0.5% (*w/v*) yeast extract (VWR, Radnor, PA, USA), 0.5% (*w/v*) bacto-peptone (VWR, Radnor, PA, USA), 0.115% (*w/v*) citric acid monohydricum (POCH, Gliwice, Poland), 0.27% (*w/v*) Na_2_HPO_4_ (POCH, Gliwice, Poland), 0.05% (*w/v*) MgSO_4_·7H_2_O (POCH, Gliwice, Poland), and 1% (*v/v*) ethanol (Chempur, Piekary Slaskie, Poland). Subsequently, the bacteria were removed from the cellulose by shaking. To obtain 14 mm biocellulose (BC) discs, 1 mL of H-S medium was poured into the wells of a 24-well plate (Wuxi Nest Biotechnology, Jiangsu, China). The wells were inoculated with the released bacteria and incubated for 7 days/28 °C. Afterwards, the BC discs were taken out, cleansed with 0.1 M NaOH (Chempur, Piekary Slaskie, Poland) at 80 °C, and rinsed with double-distilled water until neutral pH was reached. Finally, the BC discs were sterilized in an autoclave. Six BC discs were weighed, dried for 24 h at 37 °C, and weighed again. The average volume of water in discs was approximately 0.76 g. The BC discs were soaked with 1 mL of EOs or saline (positive control) or 96% (*v/v*) ethanol (for the reference strains only) and incubated for 24 h at 4 °C. The concentration of substances absorbed into the BC discs was calculated by the formula:

Compound concentration (%) = [EV/((WBC − DBC) + EV)] ∗ 100

EV—a volume of essential oil (mL).

WBC—the weight of wet BC disc (g).

DBC—the weight of dry BC disc (g).

Once the plugs were covered with biofilm (Plate 2), they were placed in the empty agar hollows of Plate 1, and 120 µL of TSB (Tryptic Soy Broth medium, Biomaxima, Lublin, Poland) was added to fill up the hollows’ space. The EOs/saline/ethanol-containing BC discs were placed on the top of the wells of the Pate 1. The plate was sealed with tape and incubated for 24 h/37 °C. 

#### 4.8.3. Viability Measurement

As incubation was completed, the BC discs and the medium over the biofilm were removed. A total of 1 mL of 0.1% (*w/v*) solution of tetrazolium chloride (TTC, 2,3,5-triphenyl-tetrazolium chloride, AppliChem Gmbh, Damstadt, Germany) in TSB was added for 2 h (37 °C) (Plate 3). Next, the solution was gently removed, and 1 mL of methanol (Stanlab, Lublin, Poland): acetic acid (Chempur, Piekary Slaskie, Poland) mixture (9:1) was added. The plate was shaken for 30 min/400 rpm at RT. From each well, three samples for 100 µL were transferred to 96-well plates (Jet Bio-Filtration Co. Ltd., Guanzhou, China), and absorbance was measured at 490 nm with a spectrophotometer (Multiskan Go, Thermo Fisher Scientific, Vantaa, Finland). Each EO and control were tested in six replicates. Compared to the biofilms treated with NaCl, reduction in biofilm metabolic activity was defined as a percentage.

### 4.9. Evaluation of Antibiofilm Activity of All Non-Emulsified EOs’ Volatile Fractions Measured with AntiBioVol Assay (Antibiofilm Activity of Volatile Compounds)

AntiBioVol test was performed as demonstrated in our previous paper [50]. The following steps of the experiment were similar to the modified A.D.A.M. assay and were conducted as follows:

#### 4.9.1. Biofilm Plugs Preparation

The first step of the experiment was performed similarly as in the modified A.D.A.M. methodology, but the wells of a 24-well plate (Plate 1) were filled with the BHI agar to the full. Moreover, one part of the divided plugs was replaced to the agar hollows of Plate 1, and the plate was kept at 8 °C. The second part was used for biofilm culturing (Plate 2). The same strains as in the modified A.D.A.M. assay were tested.

#### 4.9.2. Treatment with EOs

In the next step of the experiment, the biofilm plugs were gently transferred to Plate 1 and put on the biofilm-free plugs. All tested, undiluted essential oils were poured into a separate 24-well plate (later referred to as Plate 3) in volume 0.5 mL. Plate 1 was put upside down on Plate 3 (the agar wells were set directly above the EOs wells). The plates were sealed with tape and incubated at 37 °C for 24 h. For growth control, a 0.9% solution of NaCl (Stanlab, Lublin, Poland) was applied instead of EOs. Furthermore, the antimicrobial activity of 96% (*v/v*) ethanol (Stanlab, Lublin, Poland) was tested against the reference strains.

#### 4.9.3. Viability Measurement

After the exposure to the tested EOs, the upper plugs were gently transferred to a fresh 24-well plate (Plate 4), poured over with 2 mL of 0.1% (*w/v*) solution of tetrazolium chloride TTC (2,3,5-triphenyl-tetrazolium chloride, AppliChem Gmbh, Damstadt, Germany) in TSB and incubated for 2 h/37 °C. The solution was gently aspirated, and 2 mL of methanol (Stanlab, Lublin, Poland) and glacial acetic acid solution (Chempur, Piekary Slaskie, Poland) (9:1 ratio) was added. The plate was agitated at RT for 30 min/400 rpm using a shaker (Mini-shaker PSU-2T, Biosan SIA, Riga, Latvia). Three samples for 200 µL were transferred to 96-well plates (Jet Bio-Filtration Co. Ltd., Guanzhou, China) from each well, and absorbance measurement was performed at 490 nm wavelength using a spectrophotometer (Multiskan Go, Thermo Fisher Scientific, Vantaa, Finland). Each EO and control were examined in six replicates. Compared to the biofilms treated with NaCl, a reduction in biofilm metabolic activity (representing metabolically active cells) was defined as a percentage.

Based on the results obtained by the AntiBioVol method, two strains (the reference strain ATCC 6538 and strain referred to as SA 5) and four EOs (T-EO, E-EO, M-EO, and L-EO) were chosen for cell number quantification. The AntiBioVol assay was re-conducted, although instead of the TTC staining step, quantitative culturing was performed. For this purpose, the agar plugs treated for 24 h with volatile fractions of EOs were transferred to 1 mL of a 0.1% (*w/v*) saponin (VWR, Leuven, Belgium) solution and agitated for 30 s using a vortex mixer (Micro-shaker type 326 m, Premed, Marki, Poland). Subsequently, the serial dilutions of the suspension were cultured onto Mueller–Hinton agar (Biomaxima, Lublin, Poland) and Petri dishes (Noex, Komorniki, Poland), and then incubated for 24 h at 37 °C; finally, the CFU number was counted. The samples exposed to EOs were compared to grow control (0.9% NaCl) samples. The test was performed in three replicates.

Moreover, vapour fractions of R-EO were applied against *S. aureus* ATCC 6538 biofilm and analysed using 3D confocal microscopy. The data applied for volumetric visualization was obtained using SP8 confocal microscope (Leica Microsystems, Wetzlar, Germany) and processed using ImageJ (National Institutes of Health, Bethesda, MD, USA).

### 4.10. Fluorescence Microscopy of Biofilms Visualised with Use of LIVE/DEAD Staining

Antibiofilm activity of liquid fractions of thyme oil (T-EO) emulsion against *S. aureus* ATCC 6538 strain was examined using a fluorescence microscope (Etaluma lumascope 600, San Diego, CA, USA). Biofilm culturing and treatment with thyme oil emulsions in concentrations (*v/v*): 0.4%, 0.2%, 0.1%, 0.02%, 0.01%, and 0.006% was carried out as described in the MBEC assay. The cells exposed to 0.1% octenidine and 2% phenoxyethanol solution (Octenisept, Schulke, Wien, Austria) were used as the negative control, whereas untreated cells were used as a positive control. Filmtracer™ LIVE/DEAD™ Biofilm Viability Kit (Thermo Fischer Scientific, Waltham, MA, USA) prepared according to manufacturer’s instruction was applied as a dye to assess membrane integrity. A total of 10 µL of the reagent was added to each well of a 96-well plate (Jet Bio-Filtration Co. Ltd., Guanzhou, China) for 15 min (RT, darkness). Next, the cells were washed once with 200 µL of double-distilled water, and the plate was dried for 15 min at 37 °C. Biofilms were then analysed using a fluorescence microscope Etaluma 600 (an object lens with magnification 20×).

### 4.11. Analysis on the Size of EOs Emulsion Droplets

The analysis on size of emulsion’s droplets (the hydrodynamic diameter) were performed by dynamic light scattering (DLS) method using Zetasizer Nano ZS ZEN3600 device (Malvern Instruments, Malvern, UK). The 1000× diluted samples of 1 mL volume were introduced to disposable polystyrene cuvettes, equilibrated at 25 °C and determined with the detection angle of 173°. Data were acquired in automatic mode. The results were presented in the form of average droplets’ diameter (nm), and droplets size distribution.

## Figures and Tables

**Table 1:** Mean diameters of inhibition zones [mm]/mean radii of zones of partial growth inhibition (mm) (bolded values) after treatment with liquid fractions of EOs. T-EO—thyme oil, TT-EO—tea tree oil, B-EO–basil oil, R-EO—rosemary oil, E-EO—eucalyptus oil, M-EO—menthol mint oil, L-EO—lavender oil.

Zones of Growth Inhibition (mm) after Treatment with Liquid Fractions of EOs							
Strain	T-EO	TT-EO	B-EO	R-EO	E-EO	M-EO	L-EO
2	56 (±9.29)	8 (±6.66)	8 (±1.15)	41 (±0.58)	**0 (±0.00)/4.5**	15 (±2.65)	9 (±1.00)
4	57(±16.50)	**9 (±1.15)/3.3**	9 (±1.00)	32 (±1.53)	**0 (±0.00)/4.8**	14 (±2.31)	8 (±1.15)
5	36 (±4.04)	15 (±2.89)	10 (±1.00)	40 (±3.46)	8 (±0.58)	12 (±2.31)	9 (±1.00)
6	35 (±3.06)	15 (±1.73)	8 (±0.58)	32 (±0.58)	8 (±1.15)	11 (±1.53)	12 (±2.65)
7	55 (±13.87)	**20 (±10.79)/2.0**	17 (±0.58)	45 (±0.00)	**0 (±0.00)/6.7**	13 (±1.73)	9 (±1.15)
10	61 (±2.31)	14 (±1.15)	9 (±2.31)	35 (±0.58)	**0 (±0.00)/4.7**	12 (±1.53)	9 (±0.58)
26	43 (±3.79)	14 (±3.21)	14 (±2.08)	42 (±0.58)	**0 (±0.00)/5.7**	15 (±0.58)	9 (±1.00)
27	**60 (±13.86)/7.0**	17 (±3.06)	9 (±0.58)	27 (±3.06)	**8 (±0.58)/2.0**	13 (±2.00)	10 (±1.15)
28	**39 (±6.56)/4.2**	19 (±4.04)	12 (±5.29)	33 (±1.53)	**10 (±2.08)/2.5**	15 (±1.15)	12 (±2.00)
29	51 (±5.13)	13 (±1.15)	9 (±1.53)	38 (±3.21)	**3 (±5.20)/6.0**	10 (±0.00)	9 (±2.65)
32	50 (±9.87)	18 (±1.53)	13 (±1.15)	35 (±0.00)	11 (±0.58)	14 (±0.58)	10 (±0.58)
33	**56 (±1.73)/17.0**	24 (±1.53)	18 (±0.00)	51 (±1.00)	**0 (±0.00)/5.5**	**19 (±6.56)/6.5**	**14 (±2.89)/37.0**
34	40 (±6.35)	10 (±1.00)	18 (±0.58)	34 (±0.58)	11 (±1.15)	10 (±0.58)	8 (±1.00)
35	39 (±5.69)	13 (±1.53)	18 (±0.58)	43 (±1.73)	**8 (±0.58)/2.3**	14 (±3.61)	9 (±0.58)
ATCC 33591	43 (±3.06)	17 (±3.51)	9 (±0.00)	37 (±0.00)	**3 (±4.62)/4.0**	17 (±10.44)	10 (±1.00)
ATCC 6538	77(±12.58)	30(±5.00)	12 (±0.58)	14 (±5.13)	30(±0.5.8)	14 (±3.21)	10 (±0.58)

**Table 2:** Mean diameters of inhibition zones [mm]/mean radii of zones of partial growth inhibition (mm) (bolded values) after treatment with volatile fractions of EOs. T-EO—thyme oil, TT-EO—tea tree oil, B-EO—basil oil, R-EO—rosemary oil, E-EO—eucalyptus oil, M-EO—menthol mint oil, L-EO—lavender oil.

Zones of Growth Inhibition (mm) after Treatment with Volatile Fractions of EOs							
Strain	T-EO	TT-EO	B-EO	R-EO	E-EO	M-EO	L-EO
2	30 (±4.93)	0 (±0.00)	0 (±0.00)	13 (±8.66)	0 (±0.00)	11 (±1.73)	0 (±0.00)
4	32 (±1.00)	0 (±0.00)	0 (±0.00)	**0 (±0.00)/10.5**	**0 (±0.00)/9.3**	7 (±1.15)	0 (±0.00)
5	24 (±1.15)	0 (±0.00)	0 (±0.00)	**0 (±0.00)/11.5**	**8 (±6.93)/9.2**	2 (±3.46)	0 (±0.00)
6	24 (±1.15)	0 (±0.00)	0 (±0.00)	**0 (±0.00)/11.2**	**0 (±0.00)/12.3**	0 (±0.00)	0 (±0.00)
7	31 (±0.00)	0 (±0.00)	0 (±0.00)	**0 (±0.00)/13.0**	**6 (±9.81)/7.0**	2 (±4.04)	0 (±0.00)
10	31 (±1.15)	0 (±0.00)	0 (±0.00)	29 (±2.65)	27 (±1.73)	11 (±0.00)	0 (±0.00)
26	28 (±2.08)	0 (±0.00)	0 (±0.00)	**0 (±0.00)/13.5**	**15 (±13.61)/12.5**	2 (±4.04)	0 (±0.00)
27	30 (±3.06)	0 (±0.00)	0 (±0.00)	0 (±0.00)	**0 (±0.00)/7.5**	9 (±2.00)	0 (±0.00)
28	29 (±4.51)	0 (±0.00)	0 (±0.00)	29 (±2.52)	26 (±0.58)	8 (±7.00)	0 (±0.00)
29	31 (±2.08)	0 (±0.00)	0 (±0.00)	**0 (±0.00)/14.2**	**7 (±11.55)/10.5**	**0 (±0.00)/3.8**	0 (±0.00)
32	27 (±1.00)	0 (±0.00)	0 (±0.00)	**9 (±15.59)/21.5**	**0 (±0.00)/12.7**	**0 (±0.00)/5.2**	0 (±0.00)
33	**30 (±1.73)/13.2**	**0 (±0.00)/7.5**	**0 (±0.00)/13.0**	**28 (±1.15)/1.5**	**0 (±0.00)/13.8**	**0 (±0.00)/13.8**	**0 (±0.00)/22.8**
34	25 (±2.65)	0 (±0.00)	0 (±0.00)	**20 (±1.15)/2.8**	18 (±1.73)	0 (±0.00)	0 (±0.00)
35	21 (±1.15)	14 (±12.77)	0 (±0.00)	**0 (±0.00)/13.5**	**0 (±0.00)/6**	0 (±0.00)	0 (±0.00)
ATCC 33591	**37 (±0.58)/3.7**	**0 (±0.00)/5.5**	0 (±0.00)	**4 (±6.35)/10.0**	**0 (±0.00)/6.2**	12 (±4.93)	0 (±0.00)
ATCC 6538	43 (±5.03)	15 (±6.81)	0 (±0.00)	49 (±5.29)	0 (±0.00)	18 (±2.65)	0 (±0.00)

**Table 3:** Antimicrobial activity of liquid fractions of tested EOs’ emulsions in Tween 20 against planktonic (MIC (%) (*v/v*)) and biofilm cells (MBEC (%) (*v/v*)) of clinical (SA 2–SA 35) and reference (SA ATCC 6538 and SA ATCC 33591) strains of *S. aureus*. Dashes (-) indicate EOs where MIC and MBEC values were not reached in the highest concentration (25% (*v/v*) and 50% (*v/v*), respectively) of EOs applied. T-EO—thyme oil, TT-EO—tea tree oil, B-EO—basil oil, R-EO—rosemary oil, E-EO—eucalyptus oil, M-EO—menthol mint oil, L-EO—lavender oil.

	T-EO		TT-EO		B-EO		R-EO		E-EO		M-EO		L-EO	
Strain	MIC (%)	MBEC (%)	MIC (%)	MBEC (%)	MIC (%)	MBEC (%)	MIC (%)	MBEC (%)	MIC (%)	MBEC (%)	MIC (%)	MBEC (%)	MIC (%)	MBEC (%)
2	0.05	0.05	0.1	1.6	1.6	25	0.4	12.5	1.6	-	0.05	50	0.2	-
4	0.05	0.05	0.2	1.6	1.6	50	0.4	6.3	0.8	-	0.05	0.2	0.8	-
5	0.05	0.05	0.8	1.6	1.6	12.5	0.8	6.3	0.8	-	0.4	50	0.8	-
6	0.1	0.1	0.4	1.6	3.1	-	0.8	3.1	1.6	-	0.2	-	3.1	-
7	0.05	0.05	0.4	3.1	3.1	-	0.8	50	1.6	-	0.1	-	1.6	-
10	0.05	0.05	0.2	3.1	0.8	-	0.4	50	1.6	-	0.1	-	0.4	-
26	0.05	0.05	0.2	6.3	0.8	12.5	0.4	6.3	0.8	-	0.1	3.1	0.8	-
27	0.025	0.05	0.8	6.3	1.6	-	0.1	25	6.3	-	0.2	50	1.6	-
28	0.05	0.1	0.2	3.1	0.8	25	0.4	6.3	1.6	-	0.1	0.2	0.4	-
29	0.05	0.1	0.8	1.6	3.1	50	0.8	-	1.6	-	0.1	0.4	1.6	-
32	0.05	0.1	0.2	6.3	0.8	12.5	0.4	6.3	0.8	-	0.2	-	0.8	-
33	0.05	0.1	0.1	6.3	1.6	-	0.4	50	0.1	-	0.05	-	0.2	-
34	0.05	0.1	0.4	6.3	0.4	25	0.4	-	3.1	-	0.2	50	0.2	-
35	0.05	0.1	0.2	1.6	1.6	25	0.4	50	1.6	-	0.2	50	0.8	-
ATCC 33591	0.1	0.1	0.2	3.1	1.6	50	0.4	12.5	0.8	-	0.1	0.8	0.4	-
ATCC 6538	0.025	0.4	0.1	0.8	0.8	12.5	0.4	6.3	0.8	-	0.1	0.2	0.2	1.6

**Table 4:** Type and origin of clinical strains used in the study.

Wound Infection Strains		Bone Infection Strains	
MSSA	MRSA	MSSA	MRSA
SA 2	SA 26	SA 6	SA 32
SA 4	SA 27	SA 7	SA 33
SA 5	SA 28	SA 10	SA 34
	SA 29		SA 35

## Data Availability

The data presented in this study are available on reasonable request addressed to the corresponding author.

## Supplementary Materials

The following are available online at https://www.mdpi.com/article/10.3390/pathogens10091207/s1, Figure S1: Influence of different concentrations of Tween 20 [(%) (*v/v*)] on planktonic forms of *S. aureus* ATCC 6538 strain. C+ untreated cells., Table S1: Ingredients of tested EOs measured with GC-MS (Gas Chromatography Mass Spectrometry), Table S2: The MBC-B [(%) (*v/v*)] (minimal bactericidal concentration for biofilm) values of EOs emulsions.
