# Supplementary material for: The Antimicrobial and Antibiofilm In Vitro Activity of Liquid and Vapour Phases of Selected Essential Oils against Staphylococcus aureus
Source: Pathogens. 2021 Sep 17;10(9):1207. doi: 10.3390/pathogens10091207 (PMC8466273; doi:10.3390/pathogens10091207)
Supplement: Supplementary file 1 [file pathogens-10-01207-s001.zip › pathogens-1371846-supplementary.pdf]

Supplementary materials

The antimicrobial and antibiofilm i*n vitro* activity of liquid and vapour phases of selected Essential Oils against *Staphylococcus aureus*

Malwina Brożyna [1,*], Justyna Paleczny [1], Weronika Kozłowska [2], Grzegorz Chodaczek [3], Ruth Dudek-Wicher [1], Anna Felińczak [4], Joanna Gołębiewska [5], Agata Górniak [6] and Adam Junka [1,*]

[1] Department of Pharmaceutical Microbiology and Parasitology, Wroclaw Medical University, 50-556 Wroclaw, Poland; paleczny.justyna@gmail.com (J.P.); r.dudek.wicher@gmail.com (R.D.-W.)
[2] Department of Pharmaceutical Biology, Wroclaw Medical University, 50-556 Wroclaw, Poland; weronika.kozlowska@umed.wroc.pl (W.K.)
[3] Bioimaging Laboratory, Łukasiewicz Research Network—PORT Polish Center for Technology Development, 54-066 Wroclaw, Poland; grzegorz.chodaczek@port.org.pl (G.C.)
[4] Department of Organisation and Management
, Wroclaw Medical University, 51-618 Wroclaw, Poland; anna.felinczak@umed.wroc.pl (A.F.)
[5] Faculty of Medicine, Lazarski University, 02-662 Warsaw, Poland; joanna.golebiewska@lazarski.pl (J.G.)
[6] Laboratory of Elemental Analysis and Structural Research, Wroclaw Medical University, , 50-556 Wroclaw, Poland; agata.gorniak@umed.wroc.pl (A.G.)

Table S1. Ingredients of tested EOs measured with GC-MS (Gas Chromatography Mass Spectrometry). Components which are in line with Polish Pharmakopea XI standards are marked green colour, the ones which are not in line are marked red. **RI** - retention index, **RT** – retention time

A. Thyme oil

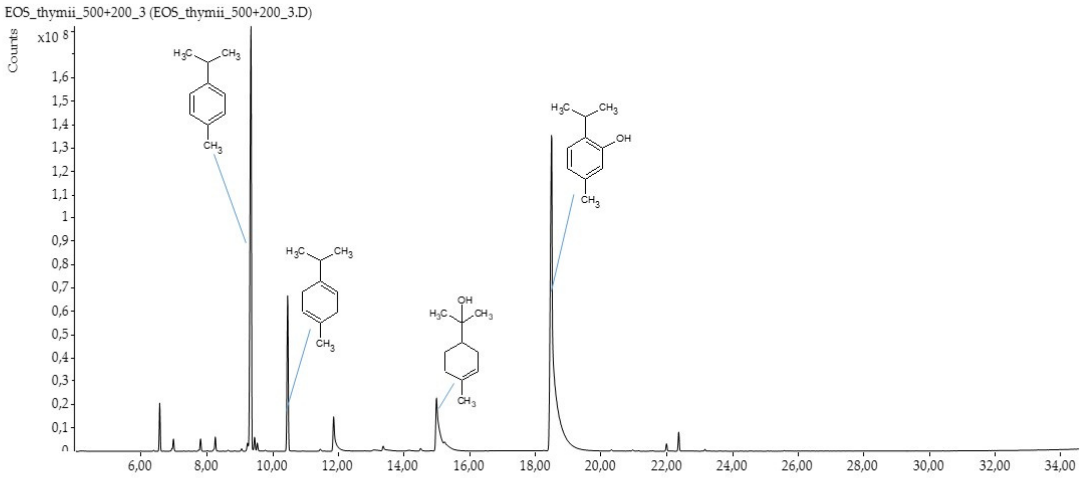

RI	RT	Compound	*T. vulgaris*
937	6.56	α-Pinene	2.20 ± 0.09
949	6.98	Camphene	0.73 ± 0.04
975	7.80	Sabinene	0.64 ± 0.03
979	8.25	β-Pinene	0.81 ± 0.05
**1025**	**9.31**	**p-Cymene**	**26.91 ± 0.99**
1028	9.45	Limonene	0.77 ± 0.04
1060	10.47	γ-Terpinene	8.60 ± 0.03
1096	11.85	Linalool	3.45 ± 0.15
1141	13.36	Camphor	0.66 ± 0.06
1189	14.99	α-Terpineol	7.84 ± 0.30
**1289**	**18.49**	**Thymol**	**44.00 ± 0.46**
1419	22.36	β-Caryophyllene	1.00 ± 0.05

Polish Pharmakopea XI ranges:

α– thujene: 0.2 per cent to 1.5 per cent
β-myrcene: 1.0 per cent to 3.0 per cent
α– terpinene: 0.9 per cent to 2.6 per cent
p- cymene: 14.0 per cent to 28.0 per cent
γ-terpinene: 4.0 per cent to 12.0 per cent
linalool: 1.5 per cent to 6.5 per cent
terpinen-4-ol: 0.1 per cent to 2.5 per cent
carvacrol methyl ether: 0.05 per cent to 1.5 per cent
thymol: 37.0 per cent to 55.0 per cent

carvacrol: 0.5 per cent to 5.5 per cent

B. Tea tree oil

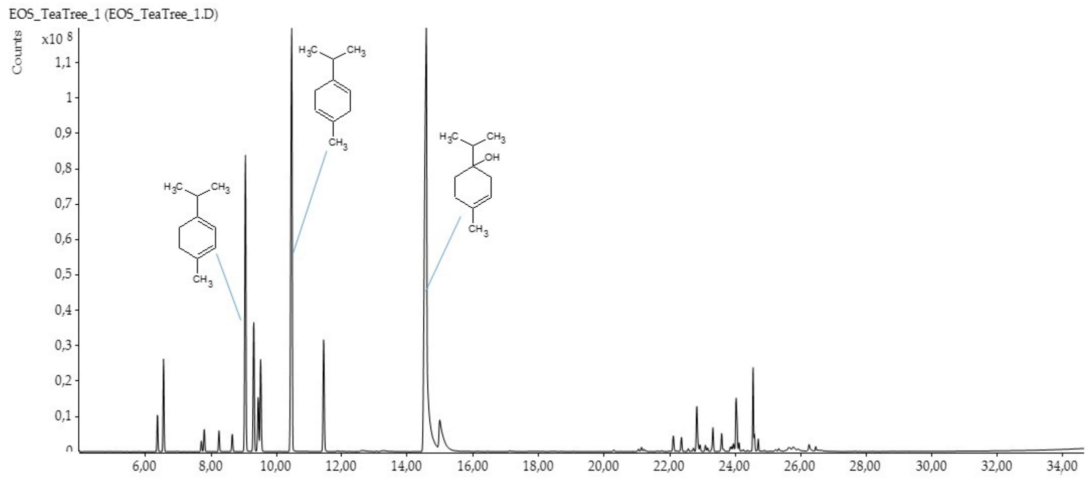

RI	RT	Compound	*M. alternifolia*
929	6.37	α-Thujene	1.11 ± 0.02
937	6.56	α-Pinene	2.85 ± 0.06
975	7.80	Sabinene	0.75 ± 0.02
979	8.25	β-Pinene	0.69 ± 0.01
1005	8.65	α-Phellandrene	0.61 ± 0.01
**1017**	**9.06**	**α-Terpinene**	**11.07 ± 0.17**
1025	9.31	p-Cymene	4.69 ± 0.07
1028	9.45	Limonene	2.08 ± 0.05
1031	9.52	1,8-Cineole	3.34 ± 0.06
**1060**	**10.47**	**γ-Terpinene**	**19.07 ± 0.27**
1088	11.44	α-Terpinolene	4.34 ± 0.06
**1177**	**14.58**	**Terpinen-4-ol**	**33.27 ± 0.79**
1189	14.99	α-Terpineol	3.26 ± 0.13
1419	22.36	β-Caryophyllene	0.53 ± 0.01
1440	22.83	Aromadendrene	1.83 ± 0.03
1460	23.32	Alloaromadendrene	0.82 ± 0.02
1496	24.03	Viridiflorene	2.35 ± 0.04
1518	24.55	β-Cadinene	2.78 ± 0.03

Polish Pharmakopea XI ranges:

α-pinene: 1.0 per cent to 6.0 per cent
sabinene: maximum 3.5 per cent
α– terpinene: 5.0 per cent to 13.0 per cent

limonene: 0.5 per cent to 4.0 per cent
cineole: maximum 15.0 per cent
γ-terpinene: 10.0 per cent to 28.0 per cent
p- cymene: 0.5 per cent to 12.0 per cent
terpinolene: 1.5 per cent to 5.0 per cent
terpinen-4-ol: minimum 30.0 per cent
aromadendrene: maximum 7.0 per cent
α– terpineol: 1.5 per cent to 8.0 per cent

C. Basil oil

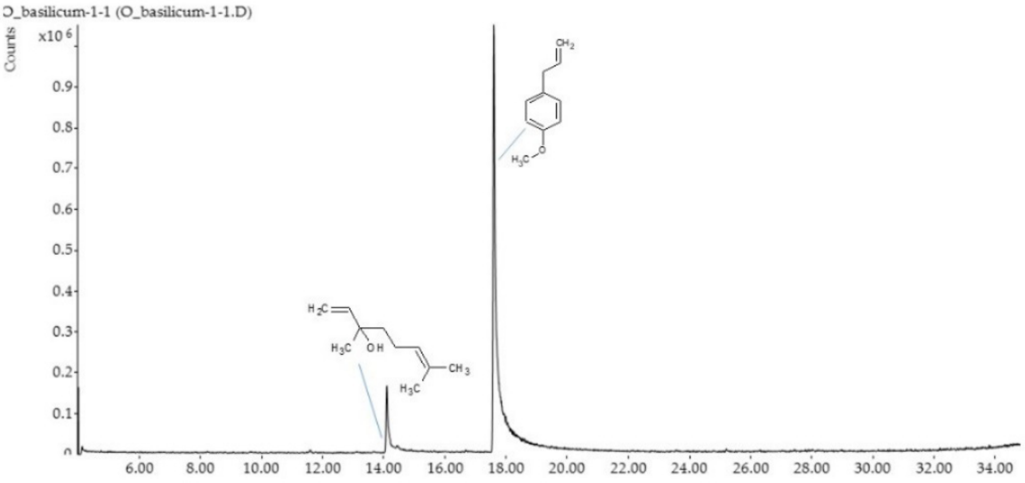

RI	RT	Compound	*O.basilicum*
**1095**	**14.08**	**Linalool**	**10.69±1.13**
**1192**	**17.59**	**Methyl chavicol [Estragole]**	**89.31±1.13**

Not included in Polish Pharmakopea XI

D. Rosemary oil

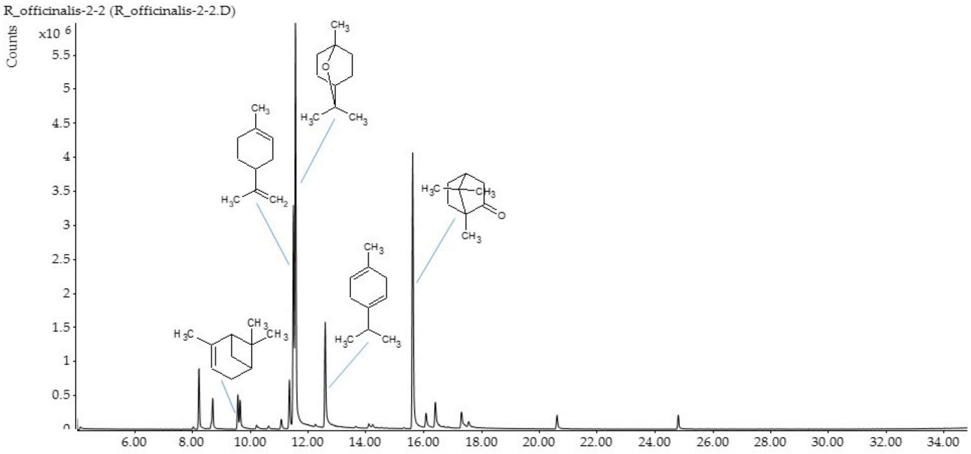

RI	RT	Compound	*R.officinalis*
896	8.22	Cyclofenchene	4.99±0.48
908	8.69	2-Bornene	3.00±0.29
924	9.57	β-Thujene	2.33±0.11
932	9.65	α-Pinene	2.58±0.28
988	10.22	Myrcene	0.38±0.14
1002	10.63	α-Phellandrene	0.23±0.07
1014	11.07	α-Terpinene	0.80±0.04
1022	11.35	o-Cymene	3.15±1.45
**1024**	**11.49**	**Limonene**	**14.26±0.99**
**1026**	**11.57**	**1,8-Cineole [Eucalyptol]**	**30.12±1.74**
1054	12.59	γ-Terpinene	8.21±0.35
**1141**	**15.64**	**Camphor**	**21.97±0.77**
1156	16.08	Isoborneol	1.53±0.06
1165	16.40	Borneol	2.69±0.09
1186	17.32	α-Terpineol	1.56±0.39
1284	20.61	Bornyl acetate	1.17±0.09
1417	24.80	Caryophyllene	0.85±0.21

Polish Pharmakopea XI ranges:

For rosemary oil, **Spanish type,** the percentages are within the following ranges:

α-pinene: 18 per cent to 26 per cent
camphene: 8.0 per cent to 12.0 per cent
β-pinene: 2.0 per cent to 6.0 per cent
β-myrcene: 1.5 per cent to 5.0 per cent
limonene: 2.5 per cent to 5.0 per cent
cineole: 16.0 per cent to 25.0 per cent
p-cymene: 1.0 per cent to 2.2 per cent

camphor: 13.0 per cent to 21.0 per cent
bornyl acetate: 0.5 per cent to 2.5 per cent
α-terpineol: 1.0 per cent to 3.5 per cent
borneol: 2.0 per cent to 4.5 per cent
verbenone: 0.7 per cent to 2.5 per cent

For rosemary oil, **Moroccan and Tunisian type**, the percentages are within the following ranges:

α-pinene: 9.0 per cent to 14.0 per cent
camphene: 2.5 per cent to 6.0 per cent
β-pinene: 4.0 per cent to 9.0 per cent
β-myrcene: 1.0 per cent to 2.0 per cent
limonene: 1.5 per cent to 4.0 per cent
cineole: 38.0 per cent to 55.0 per cent
p-cymene: 0.8 per cent to 2.5 per cent
camphor: 5.0 per cent to 15.0 per cent
bornyl acetate: 0.1 per cent to 1.5 per cent
α-terpineol: 1.0 per cent to 2.6 per cent
borneol: 1.5 per cent to 5.0 per cent
verbenone: maximum 0.4 per cent

E. Eucalyptus oil

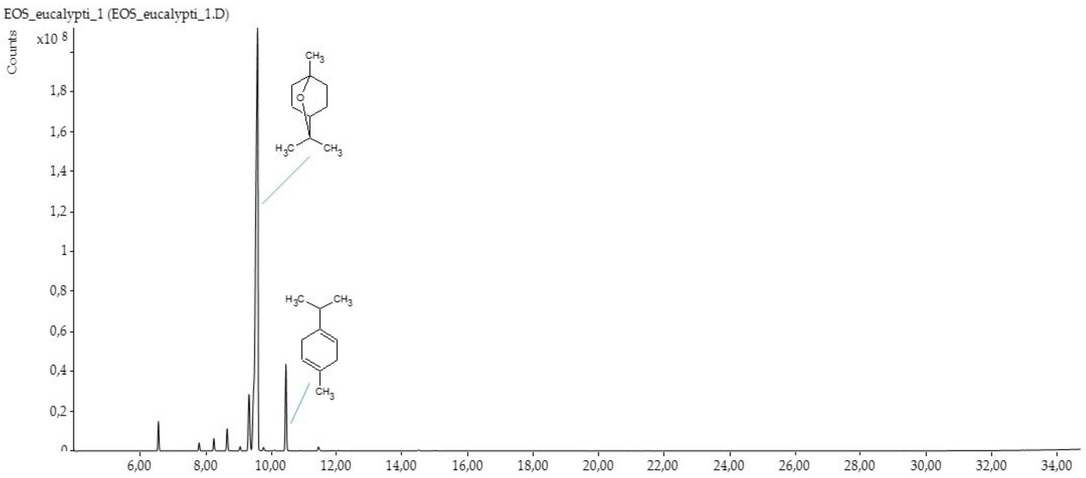

RI	RT	Compound	*E. globulus*
937	6.56	α-Pinene	2.30 ± 0.04
979	8.25	β-Pinene	1.09 ± 0.02
1005	8.65	α-Phellandrene	2.02 ± 0.02
1025	9.31	p-Cymene	6.89 ± 0.07
**1031**	**9.52**	**1,8-Cineole**	**79.10 ± 0.61**
**1060**	**10.47**	**γ-Terpinene**	**8.16 ± 0.07**

Polish Pharmakopea XI ranges

α-pinene: 0.05 per cent to 10.0 per cent
β-pinene: 0.05 per cent to 1.5 per cent
sabinene: maximum 0.3 per cent
α-phellandrene: 0.05 per cent to 1.5 per cent
limonene: 0.05 per cent to15.0 per cent
1,8-cineole: minimum 70.0 per cent
camphor: maximum 0.1 per cent

F. Menthol mint oil

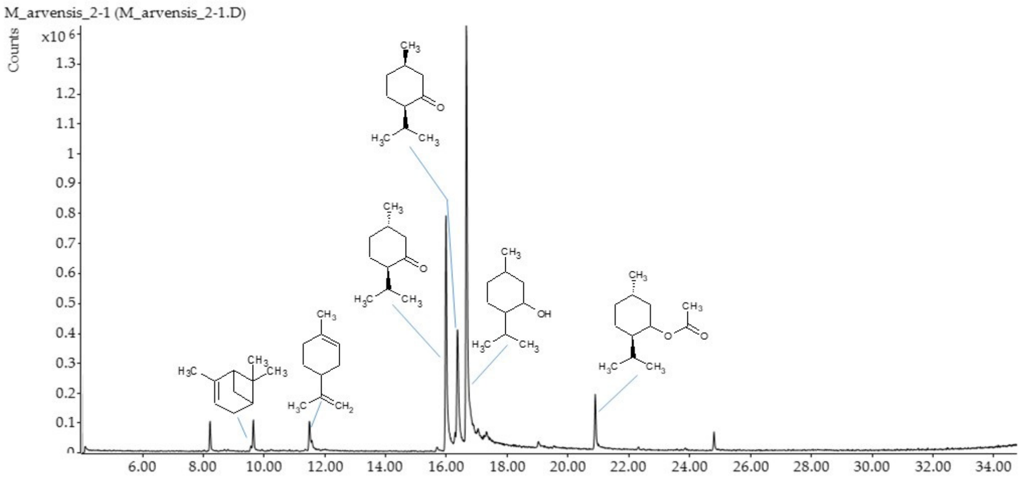

RI	RT	Compound	*M.arvensis*
896	8.22	Cyclofenchene	2.68±0.17
932	9.65	α-Pinene	3.01±0.17
1024	11.49	Limonene	3.78±0.27
**1148**	**15.98**	**Menthone**	**24.53±0.23**
**1158**	**16.36**	**Isomenthone**	**13.54±1.75**
**1167**	**16.66**	**Menthol**	**45.57±2.21**
1294	20.89	Menthyl acetate	5.61±0.36
1417	24.80	Caryophyllene	1.27±0.57

Polish Pharmakopea XI ranges:

limonene: 1.5 per cent to 7.0 per cent
cineole: maximum 1.5 per cent
menthone: 17.0 per cent to 35.0 per cent
isomenthone: 5.0 per cent to 13.0 per cent
menthyl acetate: 1.5 per cent to 7.0 per cent
isopulegol: 1.0 per cent to 3.0 per cent
menthol: 30.0 per cent to 50.0 per cent
pulegone: maximum 2.5 per cent
carvone: maximum 2.0 per cent
The ratio of cineole content to limonene content is less than 1.

G. Lavender oil

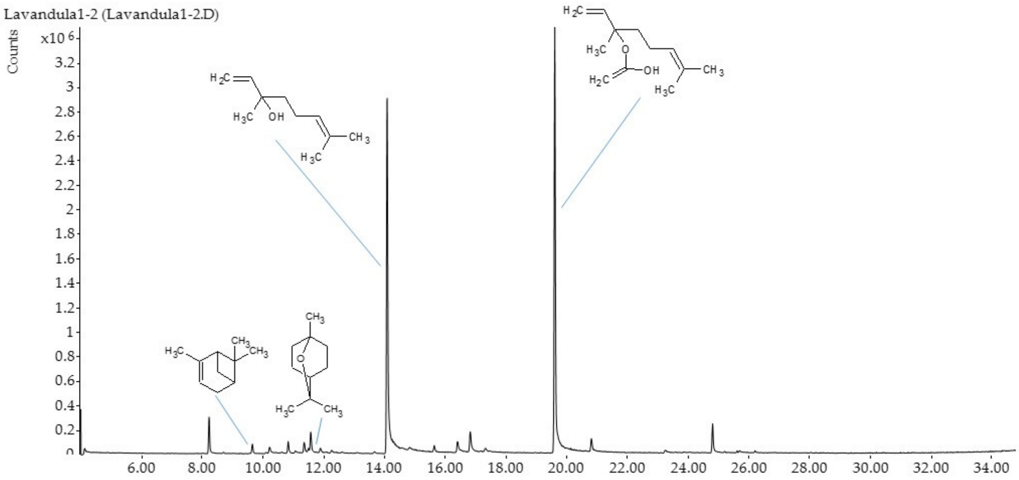

RI	RT	Compound	*L. angustifolia*
896	8.22	Cyclofenchene	3.66±0.35
932	9.65	α -Pinene	0.94±0.04
988	10.22	Myrcene	0.86±0.13
1008	10.83	3-Carene	1.19±0.12
1022	11.35	o-Cymene	1.19±0.12
1026	11.57	1,8-Cineole [Eucalyptol]	2.56±0.39
**1095**	**14.08**	**Linalool**	**37.76±1.18**
1141	15.64	Camphor	0.84±0.10

1165	16.40	Borneol	2.04±0.56
1174	16.82	Terpinen-4-ol	3.03±0.46
**1254**	**19.60**	**Linalyl acetate**	**41.13±0.40**
1288	20.81	Lavandulyl acetate	1.80±0.22
1417	24.80	Caryophyllene	3.47±0.66

Polish Pharmakopea XI ranges

limonene: maximum 1.0 per cent
1,8-cineole: maximum 2.5 per cent
3-octanone: 0.1 per cent to 5.0 per cent
camphor: maximum 1.2 per cent
linalool: 20.0 per cent to 45.0 per cent
linalyl acetate: 25.0 per cent to 47.0 per cent
terpinen-4-ol: 0.1 per cent to 8.0 per cent
lavandulyl acetate: minimum 0.2 per cent
lavandulol: minimum 0.1 per cent
α-terpineol: maximum 2.0 per cent

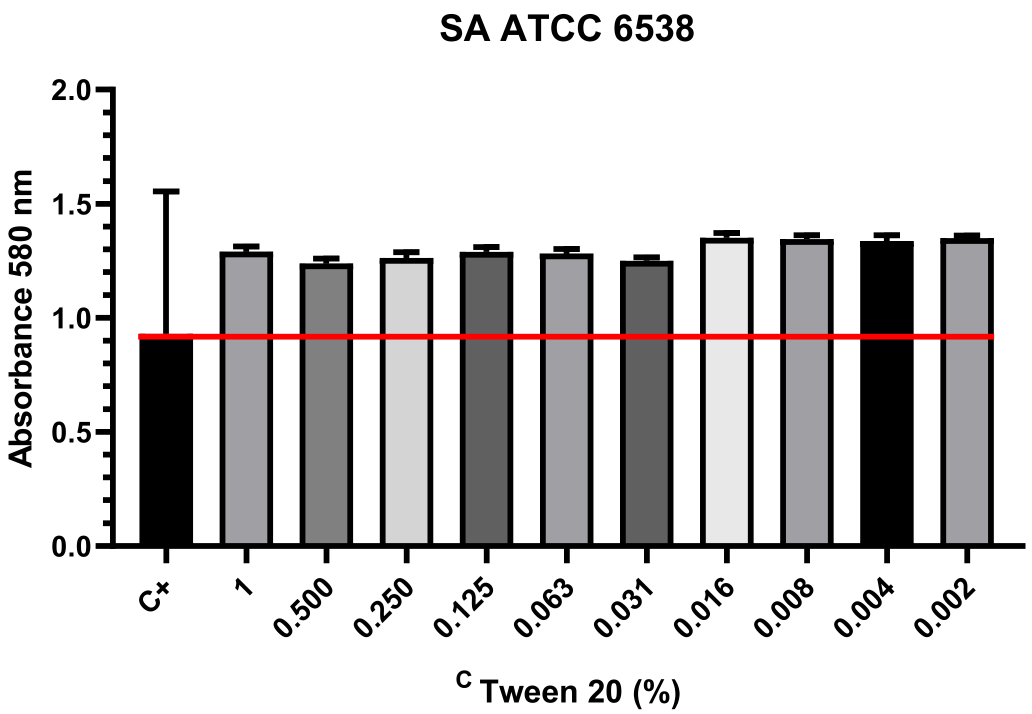

Figure S1. Influence of different concentrations of Tween 20 [(%) (v/v)] on planktonic forms of *S. aureus* ATCC 6538 strain. C+ untreated cells.

MBC-B (%)

T- EO		TT-EO		B-EO		R-EO		M-EO		L-EO	
**Strain**	**MBC-B**	**Strain**	**MBC-B**	**Strain**	**MBC-B**	**Strain**	**MBC-B**	**Strain**	**MBC-B**	**Strain**	**MBC-B**
SA 5	0.05	SA 2	25	SA 4	50	2	12.5	SA 32	50	SA 2	25
SA 6	0.1	SA 4	12.5	SA 29	50	4	6.3	SA 34	50		
SA 7	0.05	SA 5	25	SA 32	12.5	7	50	SA 35	50		
SA 10	0.05	SA 27	50	SA 34	25	27	12.5	SA ATCC 33591	25		
SA 26	0.05	SA 28	3.1	SA ATCC 33591	25	33	25	SA ATCC 6538	50		
SA 27	0.05	SA 32	6.3	SA ATCC 6538	12.5	34	25				
SA 32	0.1	SA 33	6.3			35	50				
SA 33	0.1	SA 34	6.3			SA ATCC 33591	12.5				
SA ATCC 6538	0.4	SA ATCC 33591	1.6								

Table S2. The MBC- B [(%) (v/v)] (minimal bactericidal concentration for biofilm) values of EOs emulsions. T-EO- thyme oil, TT-EO- tea tree oil, B- EO- basil oil, R-EO- rosemary oil, M-EO- menthol mint oil, L- EO- lavender oil
